# Cardiorespiratory fitness assessment using risk-stratified exercise testing and dose–response relationships with disease outcomes

**DOI:** 10.1038/s41598-021-94768-3

**Published:** 2021-07-28

**Authors:** Tomas I. Gonzales, Kate Westgate, Tessa Strain, Stefanie Hollidge, Justin Jeon, Dirk L. Christensen, Jorgen Jensen, Nicholas J. Wareham, Søren Brage

**Affiliations:** 1 MRC Epidemiology Unit, School of Clinical Medicine, Institute of Metabolic Science, University of Cambridge, Cambridge Biomedical Campus, Box 285, Cambridge, CB2 0QQ UK; 2 Yonsei University, Seoul, Republic of Korea; 3 University of Copenhagen, Copenhagen, Denmark; 4 Department Physical Performance, Norwegian School of Sport Sciences, Oslo, Norway

**Keywords:** Epidemiology, Medical research, Predictive markers, Physiology, Circulation, Respiration, Risk factors

## Abstract

Cardiorespiratory fitness (CRF) is associated with mortality and cardiovascular disease, but assessing CRF in the population is challenging. Here we develop and validate a novel framework to estimate CRF (as maximal oxygen consumption, VO_2_max) from heart rate response to low-risk personalised exercise tests. We apply the method to examine associations between CRF and health outcomes in the UK Biobank study, one of the world’s largest and most inclusive studies of CRF, showing that risk of all-cause mortality is 8% lower (95%CI 5–11%, 2670 deaths among 79,981 participants) and cardiovascular mortality is 9% lower (95%CI 4–14%, 854 deaths) per 1-metabolic equivalent difference in CRF. Associations obtained with the novel validated CRF estimation method are stronger than those obtained using previous methodology, suggesting previous methods may have underestimated the importance of fitness for human health.

## Introduction

Maximal oxygen consumption (VO_2_max) is the gold-standard measure of cardiorespiratory fitness (CRF) and a powerful predictor of all-cause and cause-specific mortality^1–3^ and morbidity^4–7^. VO_2_max is rarely directly measured in population studies due to cost and safety concerns. Alternative methods have been developed to indirectly measure VO_2_max from heart rate (HR) response to incremental submaximal exercise tests^8^. Screening procedures are generally used to exclude high-risk individuals from CRF testing, causing selection bias. Ironically, excluded individuals are more likely to experience incident disease events, making it harder to examine relationships between measured CRF and disease outcomes.

Exercise tests typically used in population studies are broadly classified as steady-state tests or ramped tests. Steady-state tests consist of several stepwise work rate (WR) increments every 4–6 min, allowing HR and VO_2_ to stabilise at each WR. Methods for estimating VO_2_max from submaximal HR response to steady-state testing are well-studied and validated^9^. Steady-state testing can be long and inefficient, however, depending on the number of WR increments and impractical for populations with low exercise tolerance^10^. Ramped tests continuously increase WR in small increments. This allows HR and VO_2_ response to be characterised over a wider range of WR values in less time and enables the rate at which WR is increased (i.e. ramp rate) to be individualised to the participant’s ability and contraindications to exercise. Ramped tests present an analytic challenge: at a given ramped WR, HR and VO_2_ values will be less than those measured during a steady-state test at an equivalent WR^11^. Thus, VO_2_max estimates from ramped tests may be biased if the HR- or VO_2_-ramp response is extrapolated from submaximal to maximal levels using methods validated for steady-state tests. Alternative methods for estimating VO_2_max from HR- or VO_2_-ramp response may be valid for common ramp rates but are insufficient for tests individualised across a wide range of ramp rates^12–14^.

We designed a ramped submaximal cycle ergometer test to assess CRF in 80,000 participants from the UK Biobank (UKB) study. The UKB-CRF test had relatively low WR and was individualised depending on presumed ability and a preliminary health risk assessment, resulting in 11 sets of protocols for men and women (22 in total). Previous attempts at estimating VO_2_max from UKB-CRF test data have assumed no difference in estimation bias between different test protocols, and some use only a small proportion of available test data^15–21^. These approaches may broadly rank individuals by CRF level, but external data are required to verify their internal validity and establish absolute validity against directly measured VO_2_max.

Here we develop and validate a novel CRF estimation method for the UKB-CRF test using exercise test data from a validation study of participants, age-, sex- and BMI-matched to the UKB sample. We first devise a hierarchical CRF estimation framework using ramped and steady-state HR response features to estimate VO_2_max. We then demonstrate the validity of VO_2_max estimates against direct measurements, apply the framework to estimate CRF in UKB participants, and conduct survival analyses to examine associations between CRF and prospective health outcomes. Finally, we compare our findings with previous methods used to estimate CRF in UKB.

## Results

### Development and validation of CRF estimation method for the UKB-CRF test

**Figure 1:**
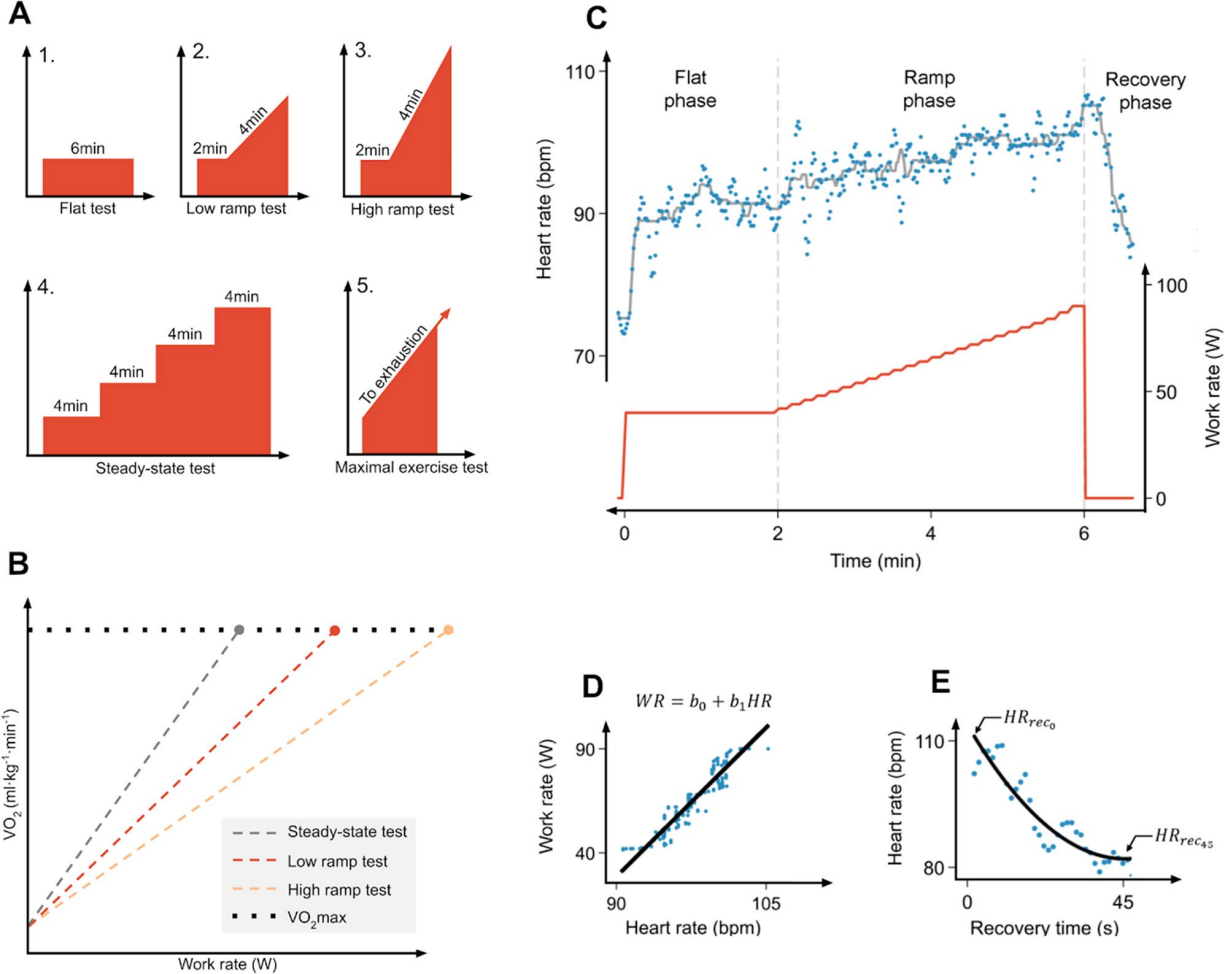
Conceptual framework and design for validation study. (**A**) Overview of the five exercise tests performed by validation study participants (3 UKB-CRF tests (flat protocol, low ramp protocol, high ramp protocol), 1 steady-state test unique to the validation study, and 1 maximal exercise test to measure VO_2_max). X-axes: Time; Y-axes: Work rate (WR). Tests were completed consecutively, and work rates were individualised according to standardised criteria (See `Experimental procedure and equipment’ in Methods). UKB-CRF test and steady-state test data were used for method development. Maximal exercise test data were withheld from method development and used for validation purposes only. (**B**) Conceptual plot of WR-to-VO_2_ response during steady-state and ramped exercise tests. VO_2_ increases linearly at a rate proportional to the rate of change in WR (i.e. ramp rate) until VO_2_max is reached (in an exhaustive test). The WR-to- VO_2_ relationship (line slope) changes depending on the ramp rate of the test. As ramp rate decreases, the WR when VO_2_max is achieved approaches the maximal WR for an exhaustive steady-state test. Note that VO_2_ is extrapolated to maximal values for demonstrative purposes, but in the validation study ramped and steady-states tests were non-exhaustive. (**C**) Exemplar HR data (blue scatter and grey line; upper panel), WR data (red line; lower panel), and test phase annotation for ramp test. (**D**,**E**) Feature extraction for ramp phase using simple linear regression model and for recovery phase using first-order exponential decay model (see Supplemental Methods).

We recruited 105 female (mean age: 54.3y ± 7.3) and 86 male (mean age: 55.0y ± 6.5) validation study participants (Supplemental Fig. 1). Participants completed a series of UKB-CRF tests and a submaximal steady-state test to characterise exercise HR response across different test protocols. VO_2_max was directly measured during an independent maximal exercise test (Fig. 1A). All participants contributed submaximal exercise test data. Some maximal exercise test data were excluded due to missing HR and VO_2_ response data (n = 25) and failure to achieve predefined maximal exercise threshold criteria (n = 33). Participant characteristics were generally similar in these subsamples.

HR response features derived from the UKB-CRF test vary with ramp rate of the assigned protocol and, if unaccounted for, will result in biased CRF estimates (Fig. 1B). HR response features may also be of poor quality or missing, a common situation in exercise testing. We addressed these issues by integrating UKB-CRF test and steady-state test HR response features (Fig. 1C–E) into a multilevel CRF estimation framework (Supplemental Table 1). The framework uses these features to estimate the HR–WR relationship that would be established if a longer steady-state test had been completed instead of the short ramped UKB-CRF test. Maximal WR is then estimated by extrapolation to age-predicted maximal HR (HRmax). This framework approach minimises bias introduced by protocol assignment of ramp rate. It also enables the application of different estimation models to different data availability scenarios. We derived several estimation models, notated as M1 through M5 in order of comprehensiveness. The highest-level model (M1) used more HR response features to estimate CRF, while lower-level models (M2 through M5) used fewer.

**Figure 2:**
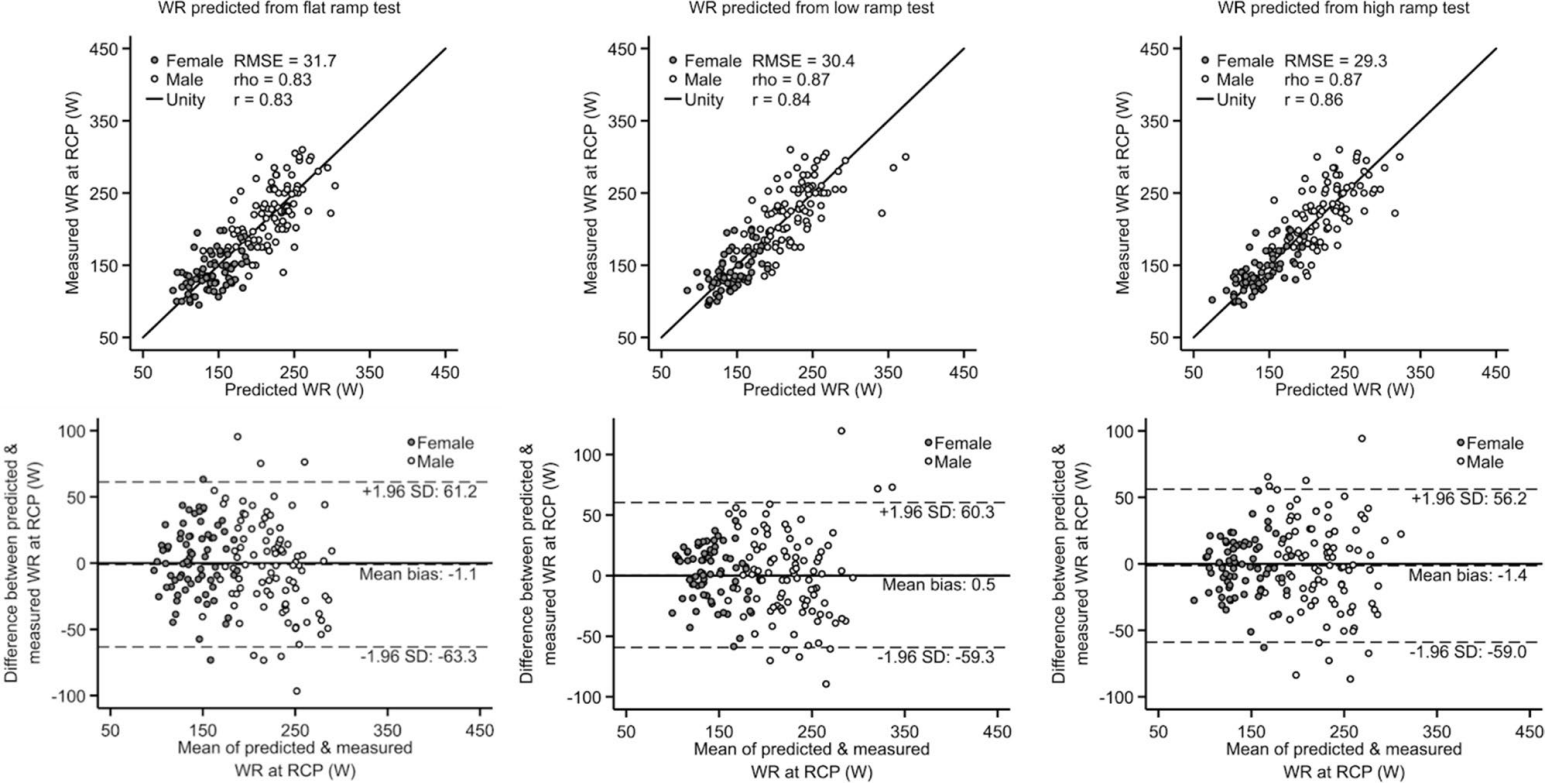
Scatterplots (top row) and Bland–Altman plots (bottom row) demonstrating agreement between work rates measured at the respiratory compensation point (RCP) and work rates estimated from flat ramp tests (left column), low ramp tests (middle column), and high ramp tests (right column) using the most comprehensive prediction equation from the multilevel framework (M1 for ramp tests; M4 for flat test). r: Pearson’s correlation coefficient, rho: Spearman’s rank correlation coefficient. RMSE: Root-mean-square error.

An accepted method for estimating VO_2_max from submaximal cycle ergometry is to estimate maximal steady-state WR from HR response and convert the estimated maximal steady-state WR to VO_2_max using the American College of Sports Medicine (ACSM) metabolic equation for cycling^9^. Before applying this equation, we verified that maximal WR estimated from the UKB-CRF test by the multilevel framework corresponded to maximal steady-state WR by comparing WR estimates with WR measured at the respiratory compensation point (RCP) from the maximal exercise test. The WR at RCP is equivalent to maximal steady-state WR and is the WR above which anaerobic metabolism is needed to sustain exercise until exhaustion. Figure 2 shows agreement with WR at RCP using the most comprehensive model for each test type (M1: ramp tests; M4: flat tests). Agreement for remaining estimation models is shown in Supplemental Table 2. Across estimation models (M1 through M5), estimated WR were strongly correlated with WR at RCP (Pearson’s *r* range 0.81–0.86) with no significant mean bias (Bias range in women: − 3.7 to 3.8 W; in men: − 5.2 to 0.1 W). Mean bias also did not differ between low and high ramped tests, but root-mean-square error was generally lower for flat tests. Correlations were higher when WR was computed using features from ramp- and recovery-phase data (models M1 through M3) compared to using only flat-phase data (models M4 and M5), although all models were relatively precise. We also compared estimated WR with WR measured at the lactate threshold (Supplemental Table 3) and WR measured at VO_2_max (Supplemental Table 4).

**Figure 3:**
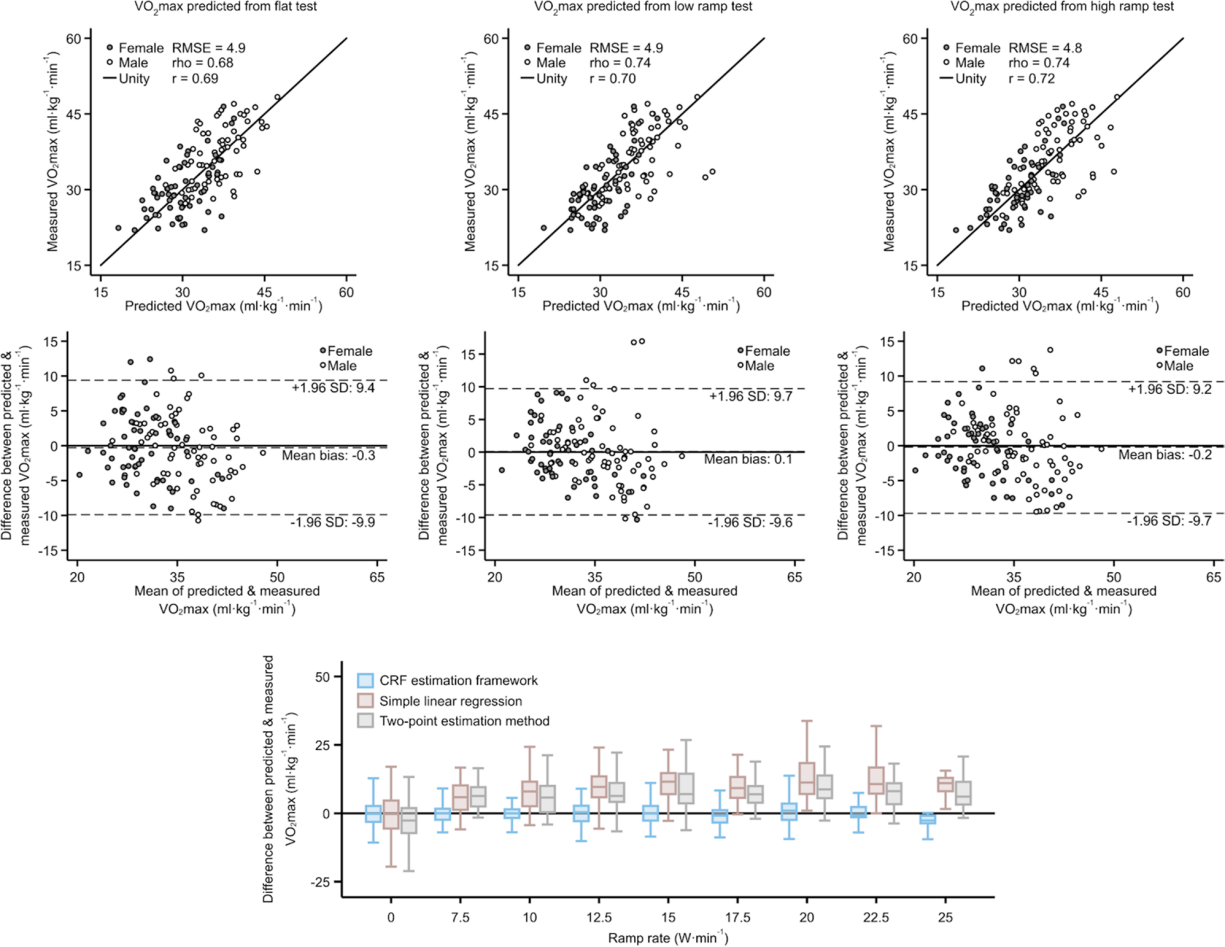
Scatterplots (top row) and Bland–Altman plots (second row) demonstrating agreement between directly measured VO_2_max and VO_2_max estimated from flat tests (left column), low ramp tests (middle column), and high ramp tests (right column) using the most comprehensive equation from the multilevel framework (M1 for ramp tests; M4 for flat test). Below these (bottom row), the box plot demonstrates agreement across all ramp rates tested using estimates from the multilevel framework, the simple linear regression method, and the two-point estimation method. r: Pearson’s correlation coefficient, rho: Spearman’s rank correlation coefficient. RMSE: Root-mean-square error.

After confirming that WR computed from the multilevel framework corresponded to WR at RCP, we estimated VO_2_max by applying the ACSM metabolic equation. We then compared these VO_2_max estimates with VO_2_max directly measured from the maximal exercise test. Figure 3 demonstrates VO_2_max agreement using the most comprehensive estimation model for each test type (M1: ramp tests; M4: flat test). Agreement for remaining estimation models is shown in Supplemental Table 5. Estimated VO_2_max was correlated with measured VO_2_max (Pearson’s r range 0.68–0.74) with no significant mean bias (Bias range in women: − 0.8 to 0.4 ml O_2_ kg^−1^ min^−1^; in men: − 0.3 to 0.3 ml O_2_ kg^−1^ min^−1^), establishing the multilevel framework’s absolute validity. We also quantified the proportion of bias emerging from uncertainty when using age-predicted versus measured HRmax in the multilevel framework (Supplemental Table 6); agreement improved only slightly when using measured HRmax. As a sensitivity analysis, we also evaluated agreement between estimated and directly measured VO_2_max in all participants with usable maximal test data and relaxing the criteria for maximal effort (n = 178). Estimated and directly measured VO_2_max were not statistically significantly different across all comparisons in this analysis.

We next addressed whether the multilevel framework would yield similar CRF estimates from different UKB-CRF test protocols completed by the same validation study participant (i.e. internal validity). Within-participant, we compared VO_2_max estimates from low and high ramp tests across estimation models M1–M3 and M5, as well as between flat tests across M4 and M5 (Supplemental Table 7). VO_2_max estimates from different UKB-CRF test protocols were highly correlated across estimation models (Pearson’s *r* range: 0.91–0.99). While mean bias was minimal across all comparisons (Bias range: − 0.6 to 0.0 ml O_2_ kg^−1^ min^−1^), some were statistically significantly different from zero mean bias. We also examined differential bias by protocol ramp rate (from 0 W min^−1^ for flat tests and 7.5–25 W min^−1^ for ramp tests), finding mean bias to be minimal across all ramp rates tested (Fig. 3, lower panel).

For method-comparison purposes, we also assessed the absolute and internal validity of a simple linear regression CRF estimation method used previously by our group and a two-point CRF estimation method used by other groups working with UKB data. These latter two methods relate exercise HR response to WR without accounting for protocol ramp rate. Both methods demonstrated overestimation bias and low precision when applied to ramped tests but had low bias when applied to flat tests (Supplemental Fig. 2).

**Figure 4:**
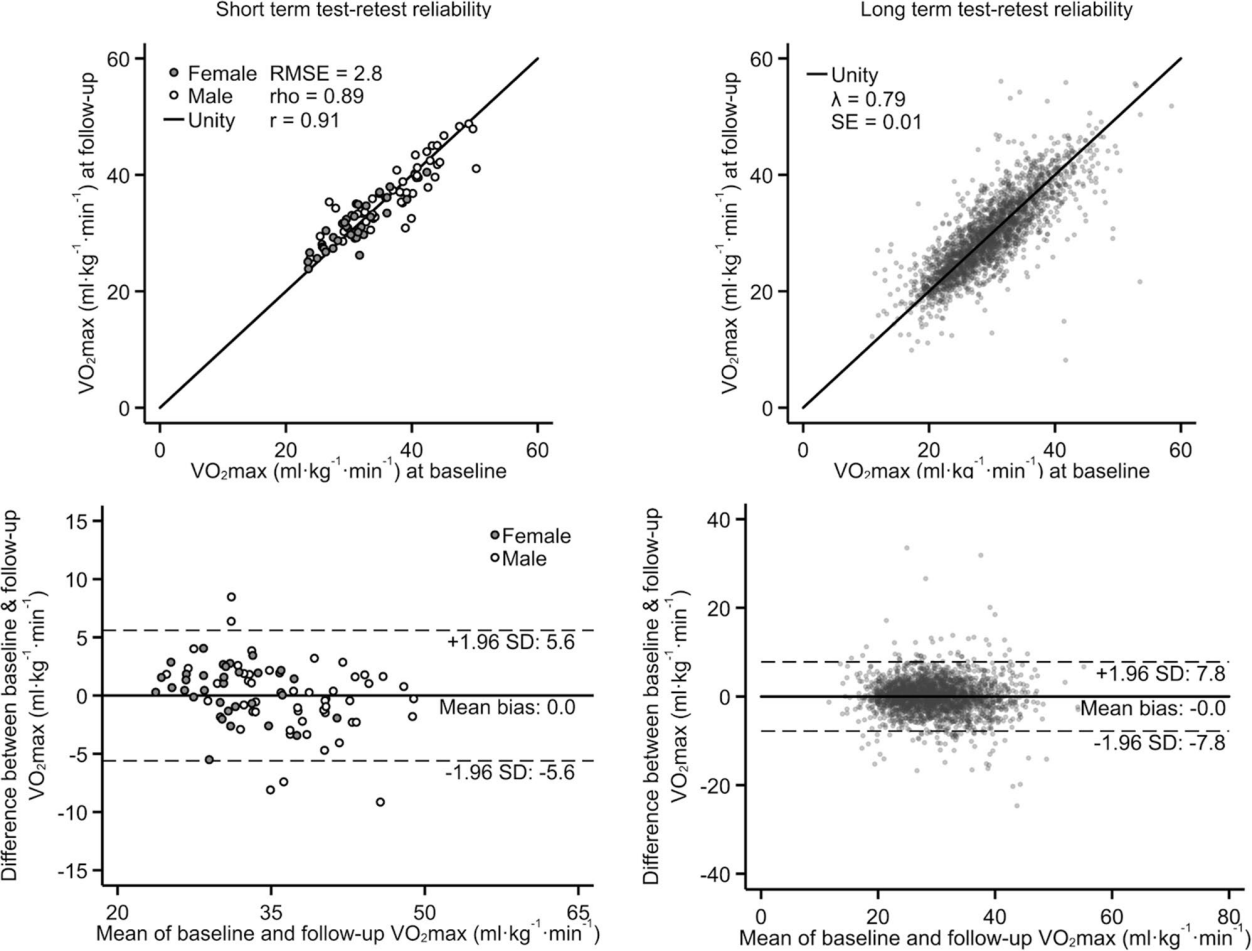
Scatterplots (top row) and Bland–Altman plots (bottom row) demonstrating short- and long-term test–retest reliability. r: Pearson’s correlation coefficient, rho: Spearman’s rank correlation coefficient. RMSE: Root-mean-square error. Lamda: Regression-dilution coefficient. SE: Standard error. Short-term reliability data are from the validation study (n = 87, follow-up ~ 10 days) and long-term reliability data are from the repeat-measures sub-study in UK Biobank (n = 2877, follow-up ~ 2.8 years).

To assess measurement consistency of the UKB-CRF test, we evaluated short-term test–retest reliability in validation study participants and long-term test–retest reliability in UKB participants (Fig. 4). In validation study participants with short-term repeat tests (within 2 weeks, n = 87), estimated VO_2_max values from the first and second UKB-CRF tests were highly correlated (r = 0.91) with no mean difference. Agreement was nearly as strong (λ = 0.79) in UKB participants with long-term repeat tests (about 2.8 years between tests, n = 2877).

### Application of VO_2_max estimation method in UKB cohort

After establishing the absolute validity, internal validity, and test–retest reliability of the multilevel framework for interpreting UKB-CRF test data, we applied the framework to estimate CRF in UKB (Supplemental Fig. 3 and Supplemental Table 8) and examined prospective associations with health outcomes. In total, 42,351 women and 37,650 men from UKB were considered in this analysis. Baseline participant characteristics are shown by sex-specific and age-adjusted CRF tertiles, across half-decade age groups, in Supplemental Table 9. Estimated VO_2_max was higher in men compared to women, and in younger versus older adults. Participants in the middle and higher CRF tertiles also had better baseline measures of heart and lung function, lower body weight, and better self-perceived health than participants in the lower tertile.

**Figure 5:**
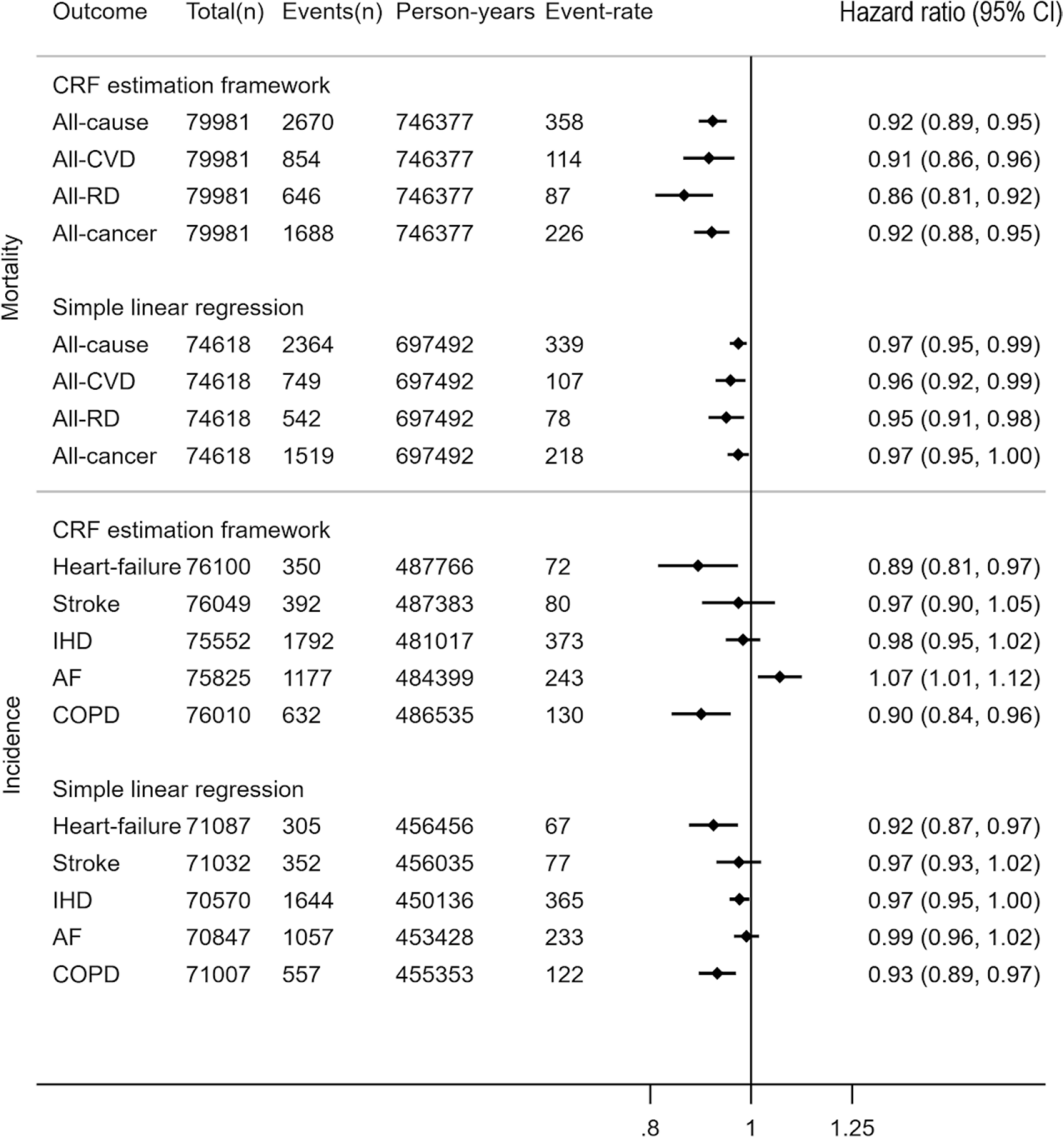
Hazard ratio and 95% confidence interval (CI) for prospective log-linear associations (Cox regression) between fatal and non-fatal outcomes in the UK Biobank with cardiorespiratory fitness in metabolic equivalents (METs, per 3.5 ml O_2_·kg^−1^ min^−1^) computed from the multilevel framework and simple linear regression methods. Event-rate per 100,000 person-years. AF: atrial fibrillation; COPD: chronic obstructive pulmonary disease; CVD: cardiovascular disease; IHD: ischaemic heart disease; RD: respiratory disease. COPD incidence mostly reflects severe COPD since only ~ 25% of cases end up in hospital. Mortality and incidence event-rates differ between fitness prediction methods owing to different inclusion criteria at the estimation level.

To examine associations between CRF and prospective health outcomes in UKB, we used Cox proportional hazards regression to estimate hazard ratios per 1-metabolic equivalent difference in CRF (METs; 1 MET = 3.5 ml O_2_ kg^−1^ min^−1^) for fatal and nonfatal events, excluding those experiencing the event in question in the first 2 years of follow-up. In total, 2670 participants died during a median 9.9 years (interquartile range 9.7–10.0) of follow-up (746,377 person-years). After adjustment for potential confounders, each 1-MET difference in CRF was associated with approximately 8% (95%CI 5–11%) lower all-cause mortality (Fig. 5); this equates to a difference in mortality of about 23% between top and bottom CRF tertiles. Associations were stronger for deaths from respiratory disease (RD; 14% lower per 1-MET, 95%CI 8–19%) and similar for deaths from cardiovascular disease (CVD; 9%, 95%CI 4–14%), and cancers (8%, 95%CI 5–12%). Higher CRF was more strongly associated with lower mortality risk in obese compared to non-obese participants (Supplemental Fig. 4).

Analyses of incident disease outcomes combined fatal and non-fatal events from hospital admissions data over 6.9 (interquartile range 6.7–7.0) years of follow-up. Risk of chronic obstructive pulmonary disease (COPD; 10% lower per 1-MET, 95%CI 4–16%) and heart-failure (11%, 95%CI 3–19%) were more strongly associated with CRF than stroke (3%, 95%CI − 5 to 10%), ischaemic heart disease (IHD; 2%, 95%CI − 2 to 5%), and atrial fibrillation (AF; − 7%, 95%CI − 1 to − 12%). Associations with CRF were not significant in obese participants for these endpoints (Supplemental Fig. 4).

For method comparison purposes, associations were also examined for CRF computed using the simple linear regression CRF estimation method. Associations were generally shallower and estimated with less uncertainty using simple linear regression, an analysis which also included fewer participants compared to the analysis using the multilevel framework.

**Figure 6:**
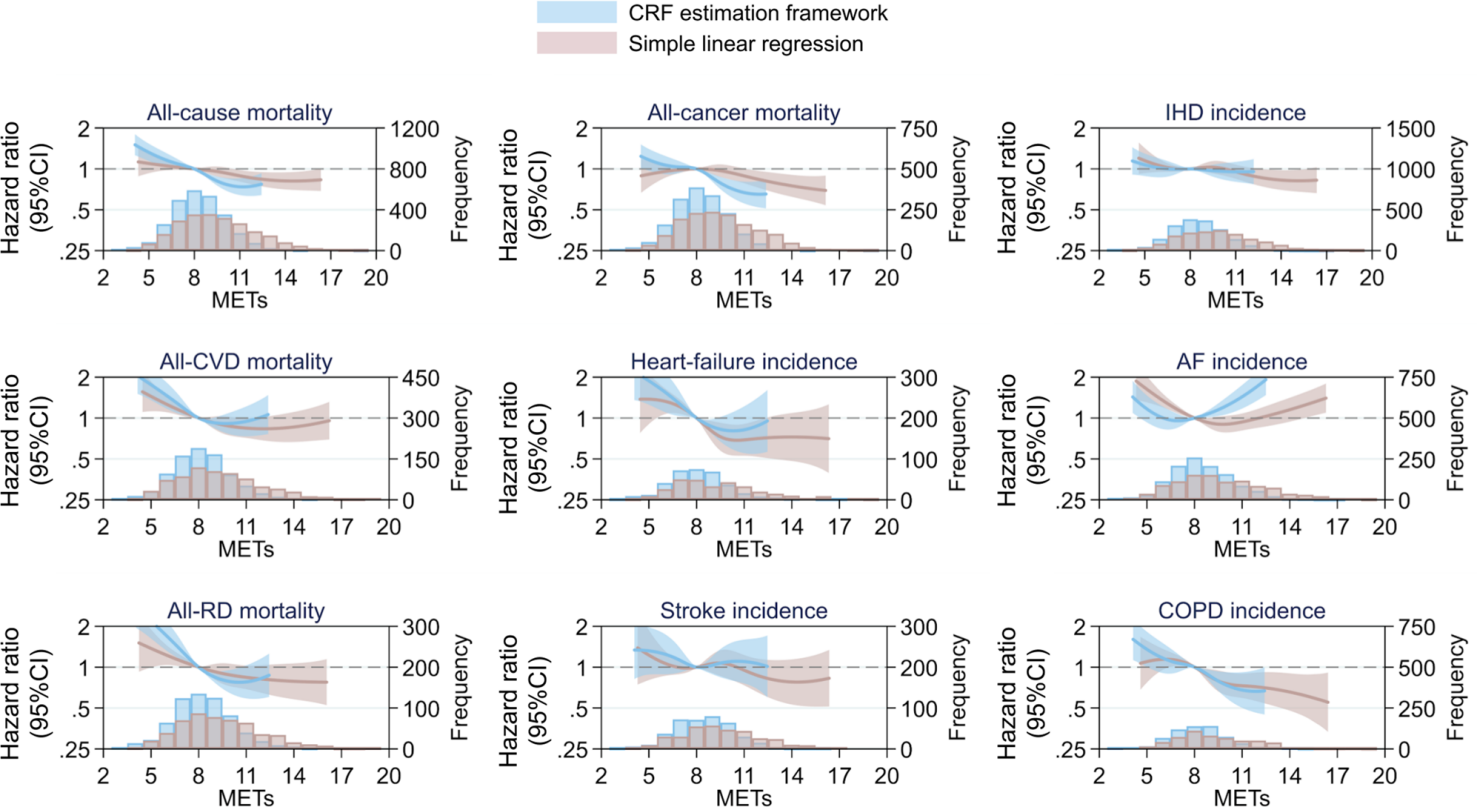
Hazard ratio and 95% confidence interval (CI) for nonlinear associations (cubic splines, Cox regression) between fatal and non-fatal outcomes in the UK Biobank with cardiorespiratory fitness in metabolic equivalents (METs, per 3.5 ml O_2_·kg^−1^·min^−1^) computed from the multilevel framework and simple linear regression methods. Hazard ratios were computed relative to a fitness reference point of 8.0 METs. AF: atrial fibrillation; COPD: chronic obstructive pulmonary disease; CVD: cardiovascular disease; IHD: ischaemic heart disease; RD: respiratory disease. Mortality and incidence counts (superimposed histograms) differ between fitness estimation methods owing to different inclusion criteria at the estimation level.

We used cubic spline regression to examine natural variation and potential nonlinearity in dose–response associations between CRF and prospective health outcomes (Fig. 6; obesity-stratified result in Supplemental Fig. 5). For method comparison purposes, separate sets of dose–response curves were modeled for CRF when computed with the multilevel framework and when using the simple linear regression method. For mortality outcomes, CRF was inversely associated with death from all causes, CVD, RD, and cancer in the CRF range of 3–11 METs. Relationships were steeper at the low end of that range and shallower at higher CRF levels. The shape of estimated CRF dose–response relationships varied considerably across incident disease outcomes. In the range of 3–8 METs, CRF was inversely associated with incidence of IHD, heart-failure, AF, stroke, and COPD, but disease associations flattened (IHD, heart failure, COPD) or became positive (AF, stroke) above 8 METs. Differences between these associations and those observed using the simple linear regression to estimate CRF were most evident at the tails of the distributions.

We performed several sensitivity analyses to determine whether health associations with CRF computed from the multilevel framework were altered by restricting the analytical sample or by using different estimation models in the multilevel framework to compute CRF. Compared to the main analysis, health associations with CRF were slightly weaker but estimated dose–response curves were qualitatively similar when the analytic sample was restricted so as to be matched with the sample used when the simple linear regression method was applied (Supplemental Figs. 6 and 7). To examine differences in health associations by estimation models in the multilevel framework, we split the analytic sample into two separate sets of analyses for those who either performed a ramp test (Supplemental Figs. 8 and 9) or a flat test (Supplemental Fig. 10). In the ramp test analysis, associations were generally stronger using more comprehensive estimation models (M1–3) compared to less comprehensive models (M5). Estimated dose–response curves were qualitatively similar for all outcomes. The association with incident AF, however, was a positive monotonic relationship at all estimation models in the ramp test subsample (event rate 217 per 100,000 person-years), rather than the U-shaped relationship found in the main analysis. In the subsample allocated a flat test (Supplemental Fig. 10), the association with incident AF was positive, non-significant, and at a higher event rate (460 per 100,000 person-years). The analysis in the flat test subsample is primarily a comparison of associations for CRF estimated by M4 and M5. Associations were generally similar between the two estimation models but non-significant due to the small sample size. We did not estimate dose–response curves in the flat test subsample for the same reason.

## Discussion

We have developed a novel multilevel CRF estimation method based on HR response to short, individualised submaximal exercise tests and applied it in one of the largest and most inclusive population-based studies of CRF. We establish the validity and reliability of this new method in an independent validation study and demonstrate advantages over other methods applied in previous population studies.

The multilevel framework method estimates VO_2_max by modeling maximal steady-state exercise capacity from HR response to the UKB-CRF test. This approach minimises CRF estimation bias that may be introduced by the UKB-CRF test individualisation process. We demonstrate that for each 1-MET difference in CRF estimated using the multilevel method, all-cause mortality was 8% lower and CVD mortality was 9% lower; associations nearly twice as strong as those estimated when using a method that did not use external data to map between ramped and steady-state exercise^15^. Improvements in CRF estimation validity and disease outcome characterisation may have broad implications for future research in UKB.

We have reported on a range of associations between CRF and prospective health outcomes in UKB, demonstrating the protective effects of CRF on all-cause and cause-specific mortality and morbidity. Our findings are largely in agreement with numerous other populations-based studies^1–3^, however associations between CRF and non-fatal incidence rates of IHD, stroke, and AF were not significant. In previous work, dose–response associations were J-shaped for stroke^22^ and U-shaped for AF^23^. Another study with longer follow-up time and more incident events reported inverse relationships between CRF and AF^24^. As for cancer mortality, we found an inverse relationship between CRF and all-cancer mortality, in agreement with previous studies^25,26^. Additional follow-up time is warranted to investigate CRF associations with site-specific cancers in UKB. Additional follow-up would, however, also increase the probability that participants may change their CRF level over time, which would dilute observed associations between a single baseline measure of CRF and health outcomes. The Cox proportional hazards model estimates the difference in hazards of the outcome in question by baseline exposure level, and if this exposure level is very stable over time, the estimated association will be a more accurate reflection of the importance of that exposure than if it were more variable. We show in this work that CRF has a long-term reliability of around 0.80, indicating good stability. However, if we were to formally apply correction for the element of instability using regression dilution bias methods, the point estimate of hazard ratios would be about 20% stronger than what we report here but with wider confidence intervals.

We have developed a novel multilevel estimation framework that optimises the validity of VO_2_max estimates from the UKB-CRF test. The key strengths of the framework are that it: (1) uses HR response features across all test phases to infer the relationship between HR and exercise intensity; (2) is flexible to the availability or absence of HR response features due to data quality issues; and (3) harmonises the inferential modeling of HR response features to the within-person invariant relationship between steady-state HR and exercise intensity. For these reasons, predicted CRF values and health associations we have presented diverge from previous reports of CRF in UKB. In a previous analysis^15^, we estimated CRF by using simple linear regression to establish the relationship between ramp test HR response and WR. This approach is currently the most common in the field but does not account for the protocol individualisation process of ramp rates used in UKB. Using external validation data, we show that this approach overestimates VO_2_max differentially by test protocol, thus limiting the ability to validly compare VO_2_max estimates from different UKB-CRF tests. Lacking protocol comparison data, previous work could only partially address the impact of this by a meta-analytic approach. Our new approach resolves this issue at the individual level and demonstrates stronger associations with all-cause and cause-specific mortality and morbidity compared to this previous non-validated method. We can have more confidence in these associations because validity of the exposure is now documented.

Other approaches also use simple linear regression, but establish the relationship between HR and exercise capacity by relating resting HR to only a single measurement of HR during the test^16,17,19–21,27–30^. HR measurement noise will greatly decrease precision of this approach, and resulting CRF estimates are still subject to bias that will differ by protocol ramp rate. Another reported approach^18,31–33^ is to use the maximally achieved WR to infer CRF. As most participants completed their test, this approach merely reflects the protocol that participants were assigned according to age, sex, body size, resting HR, and exertional chest pain risk, with the latter feature most indicative of test risk-stratification. While prospective associations of such an exposure measure with CVD endpoints do to a degree validate the stratification of risk, it is not possible to interpret these results solely as associations with CRF. At best, it is a composite score of exercise capacity and cardiac risk.

Our CRF estimation approach may also have implications for exercise prescription in clinical environments. CRF testing in UKB has demonstrated that it is safe to obtain valid VO_2_max estimates in a population setting while including some individuals with contraindications to exercise. In practice, such individuals would be prescribed a less strenuous exercise test that could contain less information about their physiological state compared to a more strenuous test. The multilevel framework approach we describe in this work for interpreting such test results yields unbiased estimates of CRF, addressing a well-recognised limitation of exercise testing^34^. Future research is warranted to investigate whether the multilevel framework can be generalised to other ramp-style exercise tests.

The strengths of our study include independent validation work for our VO_2_max estimation approach prior to estimating dose–response relationships with disease outcomes in the UK Biobank. There are also some limitations. The exercise capacity of validation study participants was slightly higher than the average capacity of UKB participants. Furthermore, the comparatively relaxed testing conditions in the validation study may not directly match those in UKB, where testing was conducted in large testing centres and where a variety of additional exposures were examined under stringent time constraints. We also did not directly evaluate the validity of UKB-CRF test protocols with ramp rates at 2.5 and 5.0 W min^−1^. Agreement for ramp rates above and below these untested rates were unbiased, however. Finally, we examined non-fatal health outcomes using only hospitalisation data; this does not necessarily capture all disease events in a given category.

## Conclusions

We have demonstrated the absolute validity, internal validity, and test–retest reliability of a novel VO_2_max estimation method for individualised ramped exercise tests that can be safely and efficiently applied in population studies. Our analytic approach uses a generalised multilevel modeling framework that bridges the gap between steady-state and ramped incremental exercise, addressing a persistent problem in exercise physiology and prescription. CRF estimated in this way is more strongly associated with mortality and other disease endpoints than previous methodology, strengthening the case for promoting CRF in the general population.

## Methods

### UKB-CRF test description

The UKB-CRF test protocol design and individualisation process are described in detail by the most recent test manual^35^. Briefly, participants were categorised into separate risk levels according to questions adapted from the Rose-Angina questionnaire. Participants with “minimal” and “small” risk completed an individualised ramp test, those with “medium” risk completed a flat test, and those with “high” risk did not complete an exercise test. Ramped tests began with a 2-min flat-phase at a single WR (30 W for females, 40 W for males) followed by a 4-min ramp-phase where WR increased continuously to a pre-specified target WR. The target WR was calculated as a risk-adjusted percentage (50% for those with “minimal” risk, 35% for “small” risk) of the maximal WR predicted from an equation derived from maximal exercise (cycle ergometer) testing data collected in the Danish Health Examination Survey 2007–2008^36^. The computed value for target WR was combined with participant sex (“F” for female, “M” for male) to notate different exercise protocols. For example, a male participant with “minimal” risk and predicted WR at VO_2_max of ~ 200 W would have a target work rate of 100 W and be individualised to UKB protocol “M100”. Flat tests consisted of a single 6-min flat-phase. Participants cycled at a 60-rpm cadence while WR and heart rate (HR) were monitored. All tests ended with a 1-min recovery-phase where participants sat quietly and motionless on the ergometer. No adverse events were reported when this test was applied in UKB.

### Validation of UKB-CRF test

#### Validation study participants

We recruited a subsample of participants from the Fenland study, a population-based study in Cambridgeshire, UK^37^, using an age-, sex- and BMI-stratified random sampling procedure (Supplemental Table 10). Exclusion criteria were: heart pacemaker; unable to walk without aid; history of angina pectoris; blood pressure greater than 180/110 mm Hg; musculoskeletal injury that would impair cycling on the ergometer; pregnancy; and currently taking cardioactive drugs (e.g. beta-blockers, aspirin). Ethical approval was obtained by the University of Cambridge Human Biology Research Ethics Committee (Ref: HBREC/2015.16). All participants provided written informed consent.

#### Experimental procedure and equipment

Validation study participants were screened according to standardised procedures used for the UKB-CRF test^35^. Then, participants completed the UKB flat test, two UKB ramped tests at different ramp rates, a steady-state test (unique to the validation study), and another ramped test (validation only) to elicit VO_2_max (Fig. 1A). Tests were conducted consecutively, separated by at least 15 min of rest, and were specified according to the test that the participant would have been assigned had they been part of UKB (see Supplemental Table 11). The target (highest) WR for the second ramped test was at least 30 W greater than the first; thus, each participant completed a “low” and “high” ramped UKB test. The steady-state test consisted of four incremental 4-min flat-phases with each WR increment ranging from 10 to 20 W. For the ramped max test, participants were fitted with a face mask to measure respiratory ventilation and gas exchange and cycled while WR increased until exhaustion. VO_2_max was considered reached if two of the following criteria were met: a respiratory exchange ratio exceeding 1.20; no VO_2_ increase despite increasing WR (< 2.5 ml O_2_ kg^−1^ min^−2^); and no HR increase despite increasing WR. During data analysis, the levelling-off criterion was confirmed by inspecting whether the first differential of HR and VO_2_ data approached zero over the last 1-min period of the maximal test.

VO_2_max was expressed as the average of the two highest VO_2_ measurements in the last forty-five seconds of the maximal exercise test. WR values were measured at VO_2_max (i.e. maximal work rate achieved on the test, WRmax), at the lactate threshold (LT), and at the respiratory compensation point (RCP). The work rate at LT was measured at the point when both ventilatory equivalent of oxygen (V_E_/VO_2_) and end-tidal pressure of oxygen (P_ET_O_2_) increased with no increase in ventilatory equivalent of carbon dioxide (V_E_/VCO_2_). The work rate at RCP was measured at the point when both V_E_/VO_2_ and V_E_/VCO_2_ increased and end-tidal pressure of carbon dioxide (P_ET_CO_2_) decreased (see Supplemental Fig. 11)^38^. Directly measured WR at LT and RCP were determined visually by three independent investigators, blinded to all other measures except the variables above needed for making direct measurements. The median value among investigators was considered the final value.

Cycling was performed on an electromagnetically-braked stationary bike (eBike ergometer, GE) while electrocardiography (ECG) was recorded using 4-lead ECG (Cardiosoft) on the forearms and a Actiwave Cardio device (CamNtech, Papworth, UK) on the chest with sampling frequency of 128 Hz. The 4-lead ECG leads were placed on the cubital fossa and ventral wrist of the left and right arms (mimicking the UKB protocol). Cycling WR were controlled by computer software. Respiratory gas measurements were conducted using a computerised metabolic system with Hans Rudolph face masks (Oxycon Pro, Erich Jaeger GmbH, Hoechberg, Germany) as validated elsewhere^39^.

All ECG signals were processed using the Physionet Toolkit implementation of the SQRS algorithm^40^, which applies a digital filter to the measured ECG and identifies the downward slopes of the QRS complexes^41^. The resulting inter-beat-intervals were converted to beats-per-minute values using the “ihr” package in the PhysioNet Toolkit, as described previously^15^. Pulmonary gas exchange data were sampled breath-by-breath. All data were linearly interpolated to derive quasi-continuous HR response and respiratory measures at 1 s time resolution. Sections of linearly interpolated HR data greater than 1 s in duration were removed prior to analysis.

#### CRF estimation framework: conceptual and modeling framework

Our approach for estimating VO_2_max from UKB-CRF test HR response is illustrated in Fig. 1B–E. Here we first describe a VO_2_max estimation method for HR response to steady-state exercise. We then adapt this method to the UKB-CRF test by using a multilevel hierarchical framework of linear models to harmonise HR response features extracted from flat and ramped UKB-CRF tests to those extracted from steady-state exercise.

##### Conceptual framework

VO_2_max can be estimated from HR response to exercise at steady-state WR increments using linear extrapolation of the submaximal HR-to-WR relationship^42^. For this approach, an individual exercises at two or more submaximal WR increments while HR is recorded. The steady-state HR response at each test increment is then regressed against WR to establish a line-of-best fit for the observed HR-to-WR relationship (W bpm^−1^). This relationship can be represented as:

1 $$\beta_{{0_{ss} }} + \beta_{{1_{ss} }} \cdot HR_{t} = WR_{t}$$

where $WR_{t}$ and $HR_{t}$ are paired measurements at several test increments, $\beta_{{1_{ss} }}$ is the linear regression slope representing the steady-state HR-to-WR relationship, and $\beta_{{0_{ss} }}$ is the intercept of that regression. The regression line is extrapolated to age-predicted maximal HR (HRmax)^43^ to estimate the maximal steady-state WR that would be achieved if the exercise test was completed to exhaustion. VO_2_max is then estimated by converting the extrapolated maximal steady-state WR value to net VO_2_ using a caloric equivalent of oxygen and adding an estimate of resting VO_2_ plus the VO_2_ required for unloaded cycling^44^.

The HR-to-WR linear extrapolation approach presents challenges when applied to ramped exercise HR response. Assuming HR and VO_2_ responses are linearly related, the principal methodological issues are^45–47^: (1) within-participant, the VO_2_-to-WR relationship and total time delay for VO_2_ response to achieve linearity after ramped exercise onset will vary across ramped tests as a function of ramp rate; (2) The ramped VO_2_-to-WR relationship decreases asymptotically with ramp rate and, as ramp rate approaches zero, becomes similar to values determined from steady-state exercise; (3) the VO_2_-to-WR relationship has high test–retest variability; and (4) the VO_2_-to-WR relationship diverges from linearity above RCP. Thus, the HR-to-WR linear extrapolation approach will induce VO_2_max overestimation bias as a function of ramp rate, demonstrate low test–retest reliability, and have poor precision if the WR computed at age-predicted HRmax is greater than the WR at RCP.

##### Modeling framework

We addressed these methodological issues by constructing a multilevel CRF estimation framework that computes a participant’s steady-state HR-to-WR relationship using features extracted from HR response to flat or ramp UKB-CRF test protocols. The framework was derived using a three-stage hierarchical linear model. The first stage equates WR computed from steady-state test HR response (Eq. 1) with WR computed from dynamic regression coefficients that vary between and within individual participants as a function of lower-stage hierarchical features. Within every *ith* individual participant, each having completed a set of *p* exercise protocols:

Stage-1 (base-stage equating steady-state test HR response with UKB-CRF flat and ramped HR response):

2 $${\beta }_{{0}_{p[ss]i}}+{\beta }_{{1}_{p[ss]i}}\cdot {HR}_{tp\left[ss\right]i}={WR}_{tp\left[ss\right]i}={\beta }_{{0}_{p\left[UKB\right]i}}+{\beta }_{{1}_{p[UKB]i}}\cdot {HR}_{tp[ss]i}$$

where (1) ${\beta }_{{0}_{p[ss]i}}$ and ${\beta }_{{1}_{p[ss]i}}$ are linear regression coefficients estimated from the steady-state protocol (*p[ss]*); (2) ${HR}_{tp[ss]i}$ is a sequence of *t* simulated steady-state HR values, equally spaced and spanning the submaximal intensity range; (3) ${WR}_{tp[ss]i}$ is a sequence of *t* steady-state WR values computed with ${\beta }_{{0}_{p[ss]i}}$, ${\beta }_{{1}_{p[ss]i}}$, and ${HR}_{tp[ss]i}$ (thus, a matrix representation of the line defined by Eq. 1); and (4) ${\beta }_{{0}_{p[UKB]i}}$ and ${\beta }_{{1}_{p[UKB]i}}$ are dynamic regression coefficients that, while unique to each UKB protocol (*p[UKB]*) and individual, converge to the values of ${\beta }_{{0}_{p[ss]i}}$ and ${\beta }_{{1}_{p[ss]i}}$ by their linkage with ${WR}_{tp[ss]i}$. ${\beta }_{{0}_{p[UKB]i}}$ and ${\beta }_{{1}_{p[UKB]i}}$ are estimated at the second stage using combinations of HR-response and protocol-based features:

Stage-2 (HR-response and protocol features extracted from flat and ramped UKB-CRF tests):

3 $${\beta }_{{0}_{p[UKB]i}}={\gamma }_{{00}_{i}}+\sum_{x \in a}{\gamma }_{{0x}_{i}}\cdot {P}_{{x}_{p[UKB]i}}$$

4 $${\beta }_{{1}_{p[UKB]i}}={\gamma }_{{10}_{i}}+\sum_{x \in a}{\gamma }_{{1x}_{i}}\cdot {P}_{{x}_{p[UKB]i}}$$

where (1) ${\gamma }_{{0x}_{i}}$ and ${\gamma }_{{1x}_{i}}$ are sets of *a* fixed regression coefficients for HR-response and protocol-level features ${P}_{{x}_{p[UKB]i}}$; and (2) ${\gamma }_{{00}_{i}}$ and ${\gamma }_{{10}_{i}}$ are the mean intercept and slope for the *ith* individual participant. ${\gamma }_{{00}_{i}}$ and ${\gamma }_{{10}_{i}}$ are estimated at the third stage:

Stage-3 (pretest participant characteristics):

5 $${\gamma }_{{00}_{i}}={\delta }_{000}+\sum_{x \in b}{\delta }_{00x}\cdot {I}_{{x}_{i}}$$

6 $${\gamma }_{{10}_{i}}={\delta }_{100}+\sum_{x \in b}{\delta }_{10x}\cdot {I}_{{x}_{i}}$$

where (1) ${\delta }_{00x}$ and ${\delta }_{10x}$ are sets of *b* fixed regression coefficients for participant characteristics ${I}_{{x}_{i}}$; and (2) ${\delta }_{000}$ and ${\delta }_{100}$ are the model-invariant intercept and slope. ${\beta }_{{0}_{p[UKB]i}}$ and ${\beta }_{{1}_{p[UKB]i}}$ can be estimated using different sets of HR-response and protocol features ${(P}_{{x}_{p[UKB]i}})$ as well as different sets of participant characteristics $({I}_{{x}_{i}})$. We leveraged this adaptability to derive five WR estimation models (notated as M1–M5; Supplemental Table 1), each using different combinations of HR response feature sets, so that our approach was robust to different data quality scenarios encountered when analysing HR response data in UKB. Additional details regarding the extraction of feature sets included in ${P}_{{x}_{p[UKB]i}}$ and ${I}_{{x}_{i}}$ are provided in Supplemental Methods.

#### Application of multilevel framework

VO_2_max was estimated from the multilevel framework by extrapolating the linear fit defined by ${\beta }_{{0}_{p[UKB]i}}$ and ${\beta }_{{1}_{p[UKB]i}}$ to age-predicted HRmax^43^ and converting the resultant maximal steady-state WR value to VO_2_max using the American College of Sports Medicine metabolic equation for cycling^9^. We also estimated WR and VO_2_max values using a simple linear regression approach^15^, a two-point estimation method^19^, and an approach for steady-state tests^15^ (see ‘Prediction of VO2max using alternative methods’ in Supplemental Materials).

#### Agreement analyses

We used Bland–Altman analysis^48^ to quantify agreement between estimated WR and VO_2_max values with those directly measured during the maximal exercise test. Correlations between estimated and directly measured values were quantified using Pearson’s *r* and Spearman’s *rho*. One-sample t-tests were performed to determine whether mean biases were statistically significantly different from zero mean bias. Estimation model precision was expressed as the root mean square error (RMSE) between estimated and directly measured values. ANOVA repeated measures were used to test differences between estimated and directly measured values across estimation models.

#### Short-term test–retest reliability

To assess short-term test–retest reliability, a subsample of 87 validation study participants completed a second UKB-CRF test within 2 weeks after main testing, identical to either the low or high ramped test at the main visit. Estimated VO_2_max values from first and second tests were compared using agreement analysis.

### Estimation of VO_2_max and associations with prospective health outcomes in UKB

#### UKB participants

The UKB is a prospective cohort study of 502,625 older adults. Baseline data collection was conducted between 2006 and 2010 where a variety of physical measurements, biological samples, and health questionnaires were administered; repeat-measures visits were conducted between 2012 and 2013. The UKB-CRF test was offered approximately 100,000 times (last 79,209 participants from baseline and 20,218 from the repeat-measures visit). The study was approved by the North West Multicentre Research Ethics Committee and participants provided written informed consent.

#### Implementation of CRF estimation in UKB

VO_2_max values were estimated in UKB participants, largely as described above for the validation study. Supplemental Fig. 12 describes specific criteria used to assign the multilevel framework estimation models for the primary analysis. Age-predicted HRmax was reduced by 20 beats-per-minute in those taking beta-blockers^49^.

#### Long-term test–retest reliability of CRF

To assess long-term test–retest reliability, we compared estimated VO_2_max values at baseline and the first follow-up test in those UKB participants with repeat tests (n = 2877, mean follow-up time 2.8 years). The follow-up UKB-CRF test protocol was re-individualised at the time of testing and therefore may have differed from the baseline protocol.

#### Health characteristics across CRF levels in UKB

Health characteristics were described across age-adjusted and sex-specific CRF categories^50^. We age-stratified the UKB cohort in half-decades as < 50, 50–54, 55–59, 60–64, and ≥ 65 years, defined CRF categories by tertiles (“lower”, “middle”, and “higher”) of estimated VO_2_max levels from each age group, and combined CRF categories from each age group to form CRF categories for the entire UKB cohort. Health characteristics were compared across CRF tertiles for men and women separately.

#### Survival analyses

Cox regression with age as the underlying timescale was used to estimate log-linear associations between estimated VO_2_max levels (in METs; 1 MET = 3.5 ml O_2_ kg^−1^ min^−1^) and mortality and incident disease outcomes. We compared prospective associations between two VO_2_max estimation approaches: the multilevel framework developed in this study and the previously described method using simple linear regression. Vital status and hospital episodes of UKB participants were established by linkage to national registry data obtained from the Health and Social Care Information Centre (now NHS Digital) for England and Wales and the Information Services Department (ISD) for Scotland. The censoring date for mortality outcomes was 31st March 2020. Censoring dates for incident disease outcomes were 31st January 2018 in England and Wales, and 30th November 2016 in Scotland. International Classification of Diseases (ICD) 10th edition (ICD-10) codes were used to define health outcomes; heart failure (I50, I11.0, I13.0, I13.2), stroke (I60-166), ischaemic heart disease (IHD; I20-I25), atrial fibrillation (AF; I48), and chronic obstructive pulmonary disease (COPD; J44). Fatal outcomes were all-cause mortality, cardiovascular disease mortality (CVD; I5-I9, I10-I89), respiratory disease mortality (RD; J00-J99), and cancer mortality (C00-97 and D00-D49).

Models were adjusted for age, sex, body weight, ethnicity, smoking status, employment status, Townsend index of deprivation, alcohol consumption, red meat intake, medication use (beta blockers, calcium channel blockers, ACE inhibitors, diuretics, bronchodialators, lipid-lowering agents, iron deficiency agents), hypertension, diabetes, and pre-baseline self-report and hospital episodes of cancer. When analysing fatal outcomes, models were additionally adjusted for pre-baseline self-report and hospital episodes of heart failure, IHD, stroke, AF, and COPD. When analysing specific incident disease outcomes (combined non-fatal and fatal), however, participants were excluded if these pre-baseline events were reported. Potential residual confounding by obesity was addressed in stratified analyses. Participants experiencing disease events in the first two years of follow-up were excluded (analysis specific). Nonlinear associations between estimated VO_2_max levels and each of the health outcomes were evaluated using a cubic spline regression model with three knots placed at the 25th, 50th, and 75th percentiles of the VO_2_max distribution. Spline models were adjusted using all covariates listed above; these models were used to estimate dose–response curves for the association between CRF and hazard ratios of the disease outcomes, using 8.0 METs as the reference exposure point.

This study complied with the items listed in the Strengthening the Reporting of Observational Studies in Epidemiology (STROBE) guidelines. All methods were performed in accordance with relevant guidelines and regulations including the Declarations of Helsinki.

### Statistical software

All analyses were performed in Stata/SE 15.1 (StataCorp, Texas, USA). Statistical significance was set at *p* < 0.05.

### Supplementary Information

Supplementary Information.
