# Author Correction: Cardiorespiratory fitness assessment using risk-stratified exercise testing and dose–response relationships with disease outcomes

**DOI:** 10.1038/s41598-023-44856-3

**Published:** 2023-10-17

**Authors:** Tomas I.Gonzales, Kate Westgate, Tessa Strain, Stefanie Hollidge, Justin Jeon, Dirk L. Christensen, Jorgen Jensen, Nicholas J. Wareham, Søren Brage

**Affiliations:** 1 MRC Epidemiology Unit, School of Clinical Medicine, Institute of Metabolic Science, University of Cambridge, Cambridge Biomedical Campus, Box 285, Cambridge, CB2 0QQ UK; 2 Yonsei University, Seoul, Republic of Korea; 3 University of Copenhagen, Copenhagen, Denmark; 4 Department Physical Performance, Norwegian School of Sport Sciences, Oslo, Norway

Correction to: *Scientifc Reports* https://doi.org/10.1038/s41598-021-94768-3, published online 28 July 2021

The original version of this article contained errors in Supplementary Information Table 1 and Table 12. In Supplementary Information Table 1, estimation models 1 and 2 (M1 and M2) rows were cut off on the right margin of the table. Additionally, in the SI Table 1 legend, ‘square root of test ramp rate’,

“W · min^−1^”.

now reads:

“W · sec^−1^”.

Similarly, in the Supplementary Information Table 12 legend, ‘square root of test ramp rate’,

“W · min^−1^”.

now reads:

“W · sec^−1^”.

The Supplementary Information file that accompanies the original Article has been corrected.

