# Supplementary material for: Cardiorespiratory fitness assessment using risk-stratified exercise testing and dose–response relationships with disease outcomes
Source: Sci Rep. 2021 Jul 28;11:15315. doi: 10.1038/s41598-021-94768-3 (PMC8319417; doi:10.1038/s41598-021-94768-3)
Supplement: Supplementary file 1 — Supplementary Information. [file 41598_2021_94768_MOESM1_ESM.pdf]

Cardiorespiratory fitness assessment using risk-stratified exercise testing and dose-response relationships with disease outcomes

Tomas I. Gonzales[1], Kate Westgate[1], Tessa Strain[1], Stefanie Hollidge[1], Justin Jeon[1,2], Dirk L. Christensen[1,3], Jorgen Jensen[1,4], Nicholas J. Wareham[1], Søren Brage[1]

1. MRC Epidemiology Unit, University of Cambridge, Cambridge, United Kingdom
2. Yonsei University, Republic of Korea
3. University of Copenhagen, Denmark
4. Department Physical Performance, Norwegian School of Sport Sciences, Norway

SUPPLEMENTAL MATERIALS

Supplemental Methods

HR response feature extraction for multilevel framework

To extract features from WR and HR response data, we applied different analysis techniques to phases within each flat, ramp, and steady-state test. Data were denoted as the tth observation in the pth exercise protocol from the ith individual participant. For ramp phase data (denoted *p[UKB]*), we used a simple linear regression model to describe the relationship between instantaneous WR and HR under ramped conditions:

$$WR_{tp[UKB]i} = b_{0_{p[UKB]i}} + b_{1_{p[UKB]i}} \cdot HR_{tp[UKB]i}$$

where $b_{0_{p[UKB]i}}$ and $b_{1_{p[UKB]i}}$ are intercept and slope parameters. If an anticipatory HR response at the flat-to-ramp phase transition was detected (defined as the difference between the median HR for the last 30s of flat phase and median HR for the initial 30s of the ramp phase exceeding 10 BPM), slope and intercept parameters were derived after excluding the initial 30s of ramp phase data.

HR dynamics during the recovery-phase were modelled using an exponential decay function:

$$HR_{tp[UKB]i} = HR_{rest_i} + e^{\mu_{0_{p[UKB]i}}} - \left(HR_{rest_i} + e^{\mu_{0_{p[UKB]i}}} - \mu_{1_{p[UKB]i}}\right) \cdot e^{-t/\mu_{2_{p[UKB]i}}}$$

where HR_{rest_i} is resting HR for participant *i*, *t* is post-exercise recovery time in seconds, and $\mu_{0_{p[UKB]i}}$,$\mu_{1_{p[UKB]i}}$, and $\mu_{2_{p[UKB]i}}$ are model parameter estimates defining HR recovery dynamics for the pth exercise protocol from the ith participant. Recovery models were solved at t = 0s and

45s to estimate HR values at the start of recovery ($HR_{rec0_{p[UKB]i}}$) and at 45s post-recovery ($HR_{rec45_{p[UKB]i}}$). Recovery HR dynamics were also characterised using a quadratic model for comparative purposes. Flat-phase data were analysed by computing the median HR value over the last minute of the test phase ($HR_{flat_{p[UKB]i}}$). For steady-state test data (denoted as *p*[*ss*]) we used a simple linear regression model to describe the relationship between WR and HR under steady-state conditions:

$$WR_{tp[ss]i} = b_{0_{p[ss]i}} + b_{1_{p[ss]i}} \cdot HR_{tp[ss]i}$$

To account for delay in the achievement of a steady-state HR at each WR increment, only HR and WR data from the last minute of each increment were used to estimate $b_{0_{p[ss]i}}$ and $b_{1_{p[ss]i}}$.

Next, in a two-step procedure, we used features extracted from WR and HR response data to estimate coefficients for a WR prediction model and several nested submodels. In the first step, intercept and slope parameters estimated from each ith participant's steady-state test ($b_{0_{p[ss]i}}$ and $b_{1_{p[ss]i}}$) were used to estimate simulated WR values that would be achieved at a set of simulated steady-state HR values ($HR_{tp[sim]i}$):

$$WR_{tp[sim]i} = b_{0_{p[ss]i}} + b_{1_{p[ss]i}} \cdot HR_{tp[sim]i}$$

where,

$$HR_{tp[sim]i} = \{80, 120, 140, 160\}$$

Thus, $WR_{tp[sim]i}$ defines a set of simulated WR values achieved under steady-state test conditions for the ith participant.

In the second step, we combined ramp-phase linear regression parameters ($b_{0_{p[UKB]i}}$ and $b_{1_{p[UKB]i}}$), HR recovery values ($HR_{rec0_{p[UKB]i}}$ and $HR_{rec45_{p[UKB]i}}$), and flat-phase median HR values ($HR_{flat_{p[UKB]i}}$) with test ramp rate ($RR_{p[UKB]i}$), participant resting HR (HR_{rest_i}) and sex (Sex_i) to construct the multilevel CRF estimation framework for predicting each participant's set of simulated steady-state WR values ($WR_{tp[sim]i}$):

Level 1 (base-stage equating steady-state test HR response with UKB CRF flat, low ramped, and high ramped HR response):

$$WR_{tp[sim]i} = \beta_{0_{p[UKB]i}} + \beta_{1_{p[UKB]i}} \cdot HR_{tp[sim]i} + r_i$$

Level 2 (HR-response and protocol features extracted from flat and ramped UKB CRF tests):

$$\beta_{0_{p[UKB]i}} = \gamma_{00_i} + \gamma_{01_i} \cdot b_{0_{p[UKB]i}} + \gamma_{02_i} \cdot HR_{rec0_{p[UKB]i}} + \gamma_{03_i} \cdot HR_{rec45_{p[UKB]i}} + \gamma_{04_i} \cdot HR_{flat_{p[UKB]i}} + \gamma_{05_i} \cdot RR_{p[UKB]i}$$

$$\beta_{1_{p[UKB]i}} = \gamma_{10_i} + \gamma_{11_i} \cdot b_{1_{p[UKB]i}} + \gamma_{12_i} \cdot HR_{rec0_{p[UKB]i}} + \gamma_{13_i} \cdot HR_{rec45_{p[UKB]i}} + \gamma_{14_i} \cdot HR_{flat_{p[UKB]i}} + \gamma_{15_i} \cdot RR_{p[UKB]i}$$

Level 3 (pretest participant characteristics):

$$\gamma_{00_i} = \delta_{000} + \delta_{001} \cdot HR_{rest_i} + \delta_{002} \cdot Sex_i$$

$$\gamma_{10_i} = \delta_{100} + \delta_{101} \cdot HR_{rest_i} + \delta_{102} \cdot Sex_i$$

where r_i is a random intercept to control for clustering of observations within participants. Features were removed in a stepwise fashion to estimate coefficients for five estimation models, each designed to accommodate different data availability scenarios. Features were also removed to maximise explained variance with the fewest degrees of freedom.

Prediction of VO_2max using multilevel framework

To predict VO_2max, work rate values were estimated using the top estimation model for a given data availability scenario (see Supplemental Figure 12) by substituting $HR_{i[ss]}$ with age-predicted maximal HR:

$$HR_{max} = 208 - 0.7 \cdot Participant\ age\ (years)$$

Then, estimated work rate values were converted to VO_2 values using the American College of Sports Medicine metabolic equation for cycle ergometry:

$$VO_2 = 1.8 \cdot 6.12 \cdot \frac{Predicted\ work\ rate}{Participant\ weight\ (kg)} + 7$$

Prediction of VO_2max using alternative methods

VO_2max values were also estimated using three alternative methods. The first method, a simple linear regression approach, was applied to "low" and "high" ramp tests completed by participants:

$$WR_{pi} = b_{0_{pi}} + b_{1_{pi}} \cdot HR_{max_i}$$

where $b_{0_{pi}}$ and $b_{1_{pi}}$ are intercept and slope parameters described previously in the ramp phase test analysis and HR_{max_i} is age-predicted maximal HR. Next, a two-point approach, is similar to simple linear regression but simplifies this approach by using participant resting HR (HR_{rest_i}) and the highest ramp phase HR value (HR_{rest_i}) and work rate value ($WR_{ramp_{pi}}$):

$$WR_{pi} = \frac{WR_{ramp_{pi}}}{HR_{ramp_{pi}} - HR_{rest_i}} \cdot \left(HR_{max_i} - HR_{rest_i}\right)$$

The last method was applied to flat tests:

$$WR_{pi} = \frac{WR_{flat_p}}{HR_{flat_{pi}} - HR_{rest_i}} \cdot \left(HR_{max_i} - HR_{rest_i}\right)$$

where W_{flat_p} is the test steady-state work rate (30W for females; 40W for males). Work rate values were converted to predicted VO_2max values using the ACSM metabolic equation for cycle ergometry.

Supplemental Figures

Supplemental Figure 1

Validation study participant characteristics across each validity subanalysis.

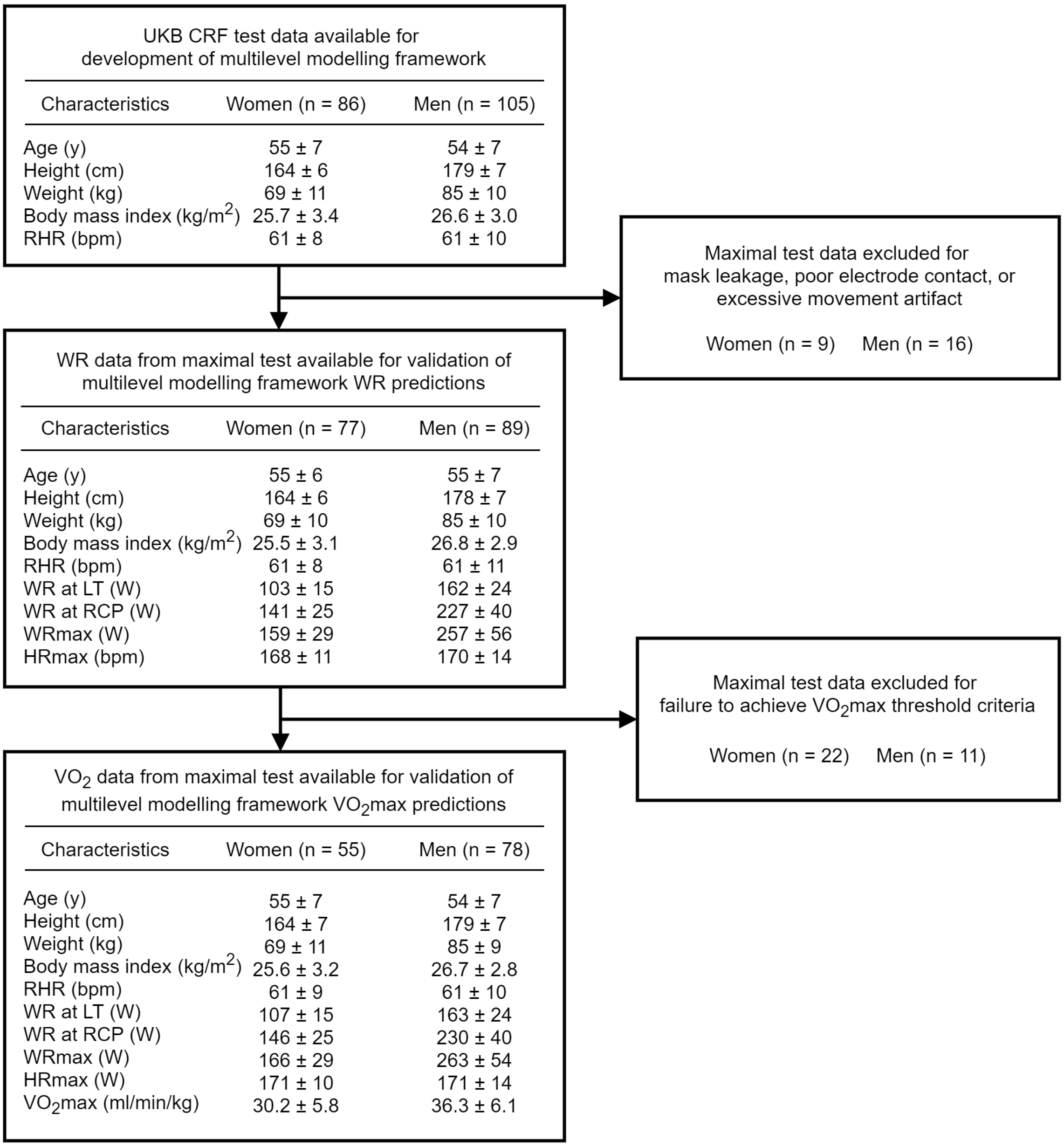

RHR: Resting heart rate, WR: Work rate, LT: Lactate threshold, RCP: Respiratory compensation point,
WRmax: Measured maximal work rate, HRmax: Measured maximal heart rate, VO_2max: Maximal oxygen consumption

Supplemental Figure 2

Scatterplots and Bland-Altman plots demonstrating agreement between directly measured VO_2max and VO_2max estimated from the flat test, low-ramp test, and high-ramp test using: A. simple linear regression; and B. 2-point estimation method. r: Pearson's correlation coefficient, rho: Spearman's rank correlation coefficient. RMSE: Root-mean-square error.

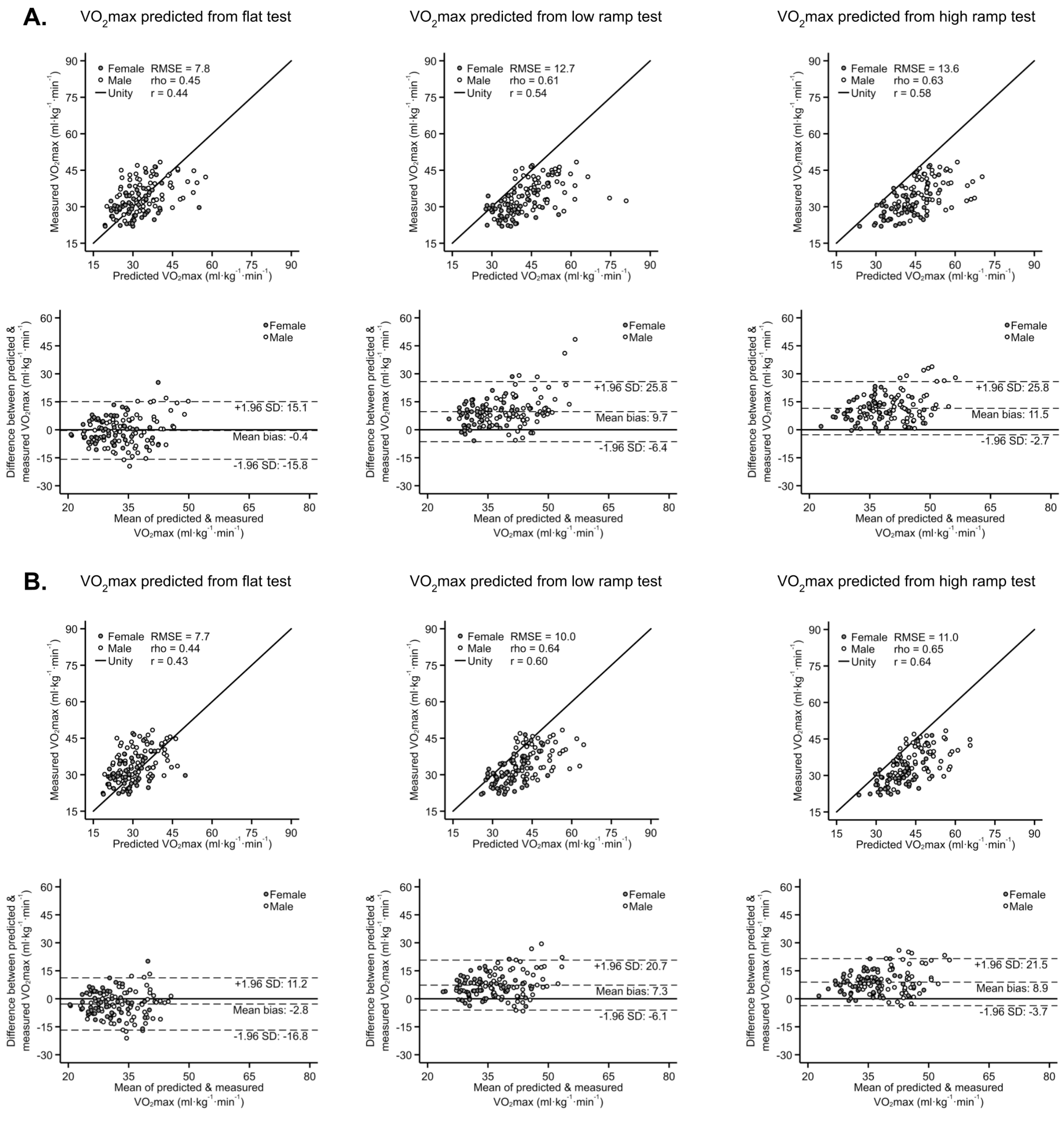

Supplemental Figure 3

Left panel: Flow diagram showing the number of participants included and excluded in the UKB cohort analysis (characteristics of included sample in Supplementary Table 8), as well as the allocation of multilevel framework estimation models (see Supplemental Figure 12 for an explanation of this process). HR responses for UKB participants were recorded as either raw ECG or “Trend” data. “Trend” data represents instantaneous HR values computed using a proprietary algorithm in the software used to record data (Cardiosoft); in some tests sessions, this is the only data available (no raw ECG). Top right panel: Differences between the subsample of UKB participants with and without a bike test and stratified by eligibility, using VO_2max estimated from resting HR within the bike test sample (VO_2max = -0.28 RHR + 6 male sex + 44, $R^2 = 0.45$, RMSE = 4.9 ml O_2/min/kg). Histograms represent frequency of target work rates for UKB CRF tests in the subsample only across risk strata. Bottom-right panel: Sensitivity analysis comparing predicted values from ECG and “Trend” data across estimation equations and within-participant, demonstrating no differences between data capture methods. ECG data were chosen preferentially over “Trend” data when both data sources were available.

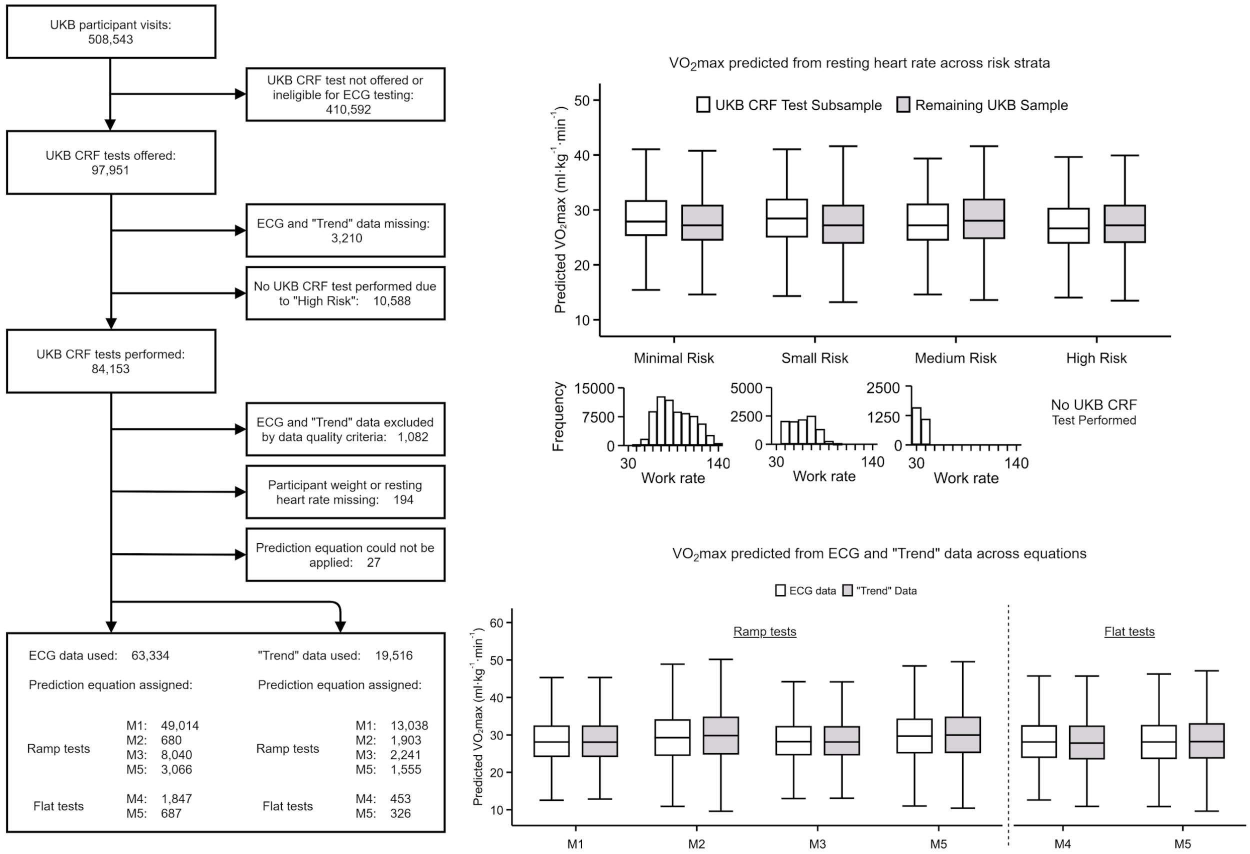

Supplemental Figure 4

Hazard ratios and 95% confidence intervals (CI) for prospective log-linear associations between fatal and non-fatal outcomes in the UK Biobank with cardiorespiratory fitness in metabolic equivalents (METs, per 3.5 ml $O_2 \cdot kg^{-1} \cdot min^{-1}$), stratified by obesity status in UKB participants. Event-rate per 100,000 person years. AF - atrial fibrillation; COPD: chronic obstructive pulmonary disease; CVD: cardiovascular disease; IHD: ischaemic heart disease; RD- respiratory disease. COPD incidence mostly represents severe COPD since only ~25% of cases end up in hospital. CRF was computed using the multilevel framework.

	Outcome	Total(n)	Events(n)	Person-years	Event-rate	Hazard ratio (95% CI)
Mortality	Non-obese					
	All-cause	62505	1928	582607	331	0.93 (0.90, 0.96)
	All-CVD	62505	555	582607	95	0.94 (0.88, 1.00)
	All-RD	62505	470	582607	81	0.88 (0.81, 0.94)
	All-cancer	62505	1242	582607	213	0.92 (0.88, 0.96)
	Obese					
	All-cause	17476	742	163770	453	0.87 (0.81, 0.93)
	All-CVD	17476	299	163770	183	0.85 (0.76, 0.95)
	All-RD	17476	176	163770	107	0.76 (0.66, 0.88)
	All-cancer	17476	446	163770	272	0.88 (0.81, 0.97)
Incidence	Non-obese					
	Heart-failure	59893	218	382942	57	0.90 (0.81, 1.00)
	Stroke	59847	287	382548	75	1.00 (0.91, 1.09)
	IHD	59523	1238	378321	327	1.00 (0.96, 1.05)
	AF	59696	789	380562	207	1.09 (1.03, 1.15)
	COPD	59819	455	381927	119	0.91 (0.84, 0.97)
	Obese					
	Heart-failure	16207	132	104824	126	0.92 (0.78, 1.08)
	Stroke	16202	105	104834	100	0.88 (0.73, 1.06)
	IHD	16029	554	102697	539	0.94 (0.86, 1.02)
	AF	16129	388	103837	374	1.00 (0.90, 1.11)
	COPD	16191	177	104608	169	0.84 (0.73, 0.98)

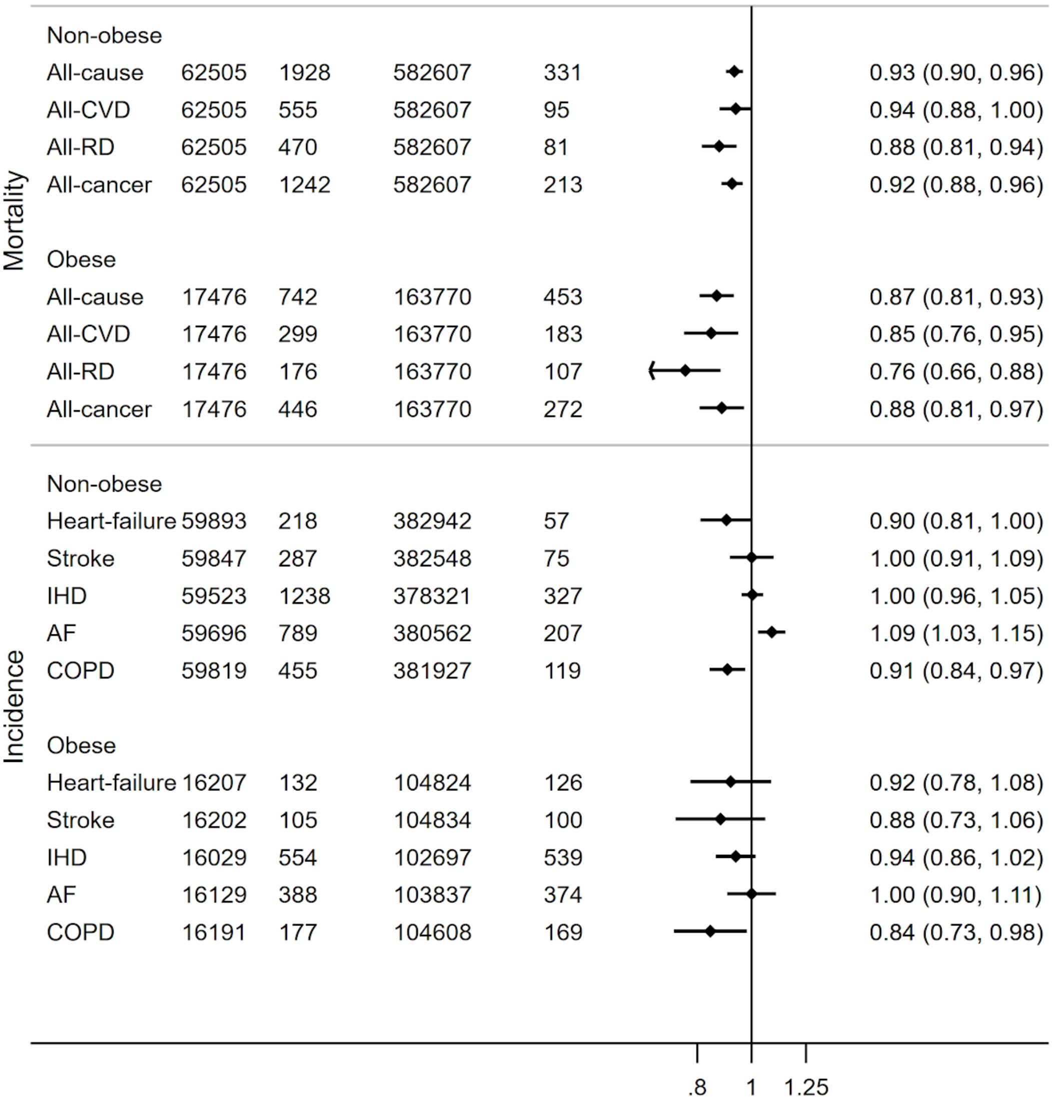

Supplemental Figure 5

Hazard ratios and 95% confidence intervals (CI) for nonlinear (cubic spline) associations between fatal and non-fatal outcomes in the UK Biobank with cardiorespiratory fitness in metabolic equivalents (METs, per 3.5 ml O2·kg^{-1}·min^{-1}), stratified by obesity status in UKB participants. Hazard ratios were computed relative to a fitness reference point of 8.0 METs. AF: atrial fibrillation; COPD: chronic obstructive pulmonary disease; CVD: cardiovascular disease; IHD: ischaemic heart disease; RD: respiratory disease. CRF was computed using the multilevel framework.

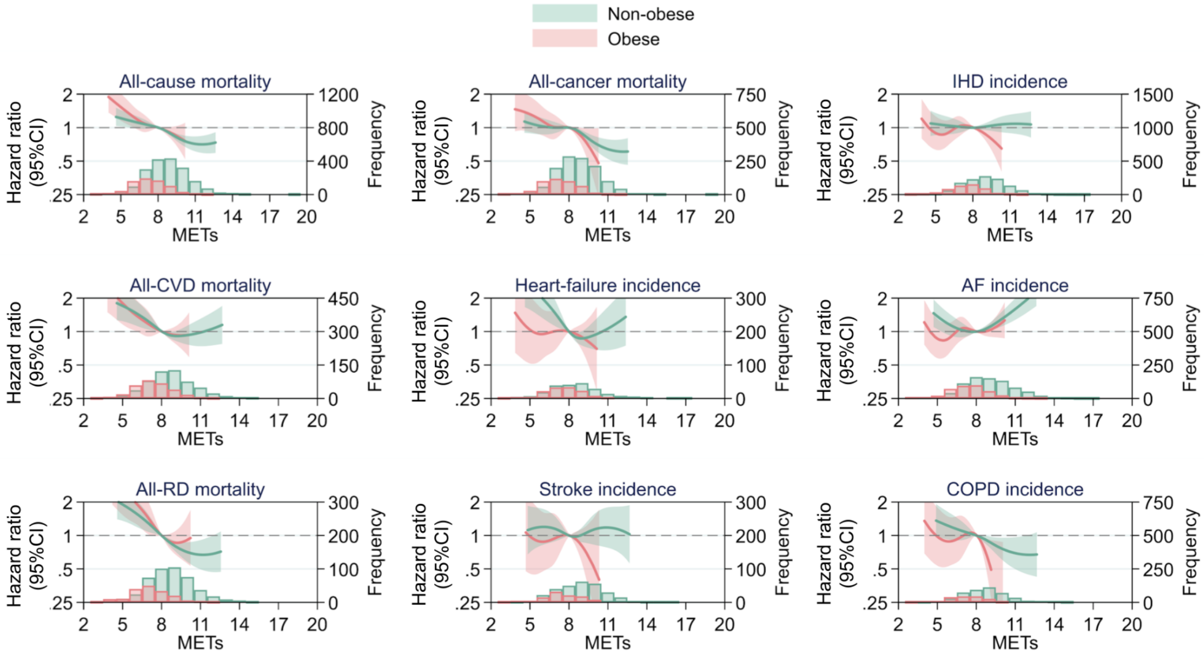

Supplemental Figure 6

Sample-matched hazard ratios and 95% confidence intervals (CI) for prospective log-linear associations between fatal and non-fatal outcomes in the UK Biobank with cardiorespiratory fitness in metabolic equivalents (METs, per 3.5 ml $O_2 \cdot kg^{-1} \cdot min^{-1}$) computed from the multilevel framework and simple linear regression methods. Event rate per 100,000 person-years. AF - atrial fibrillation; COPD: chronic obstructive pulmonary disease; CVD: cardiovascular disease; IHD: ischaemic heart disease; RD- respiratory disease. COPD incidence mostly represents severe COPD since only ~25% of cases end up in hospital. For these analyses, the analytical sample was matched between fitness estimation methods.

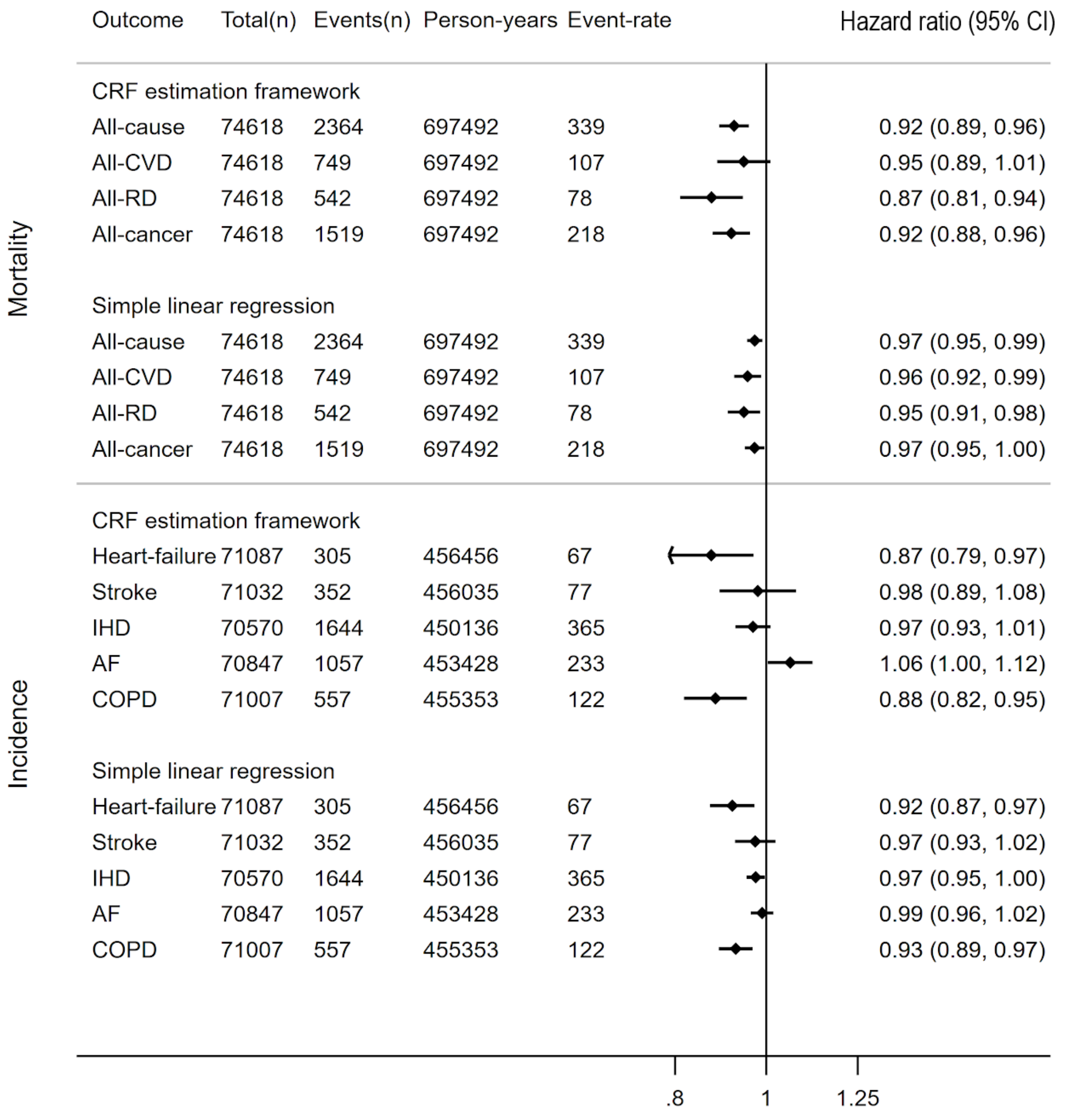

Supplemental Figure 7

Sample-matched hazard ratios and 95% confidence intervals (CI) for nonlinear associations (cubic splines, Cox regression) between fatal and non-fatal outcomes in the UK Biobank with cardiorespiratory fitness in metabolic equivalents (METs, per 3.5 ml O2·kg^{-1}·min^{-1}) computed from the multilevel framework and simple linear regression. Hazard ratios were computed relative to a fitness reference point of 8.0 METs. AF: atrial fibrillation; COPD: chronic obstructive pulmonary disease; CVD: cardiovascular disease; IHD: ischaemic heart disease; RD: respiratory disease. For these analyses, the analytical sample was matched between fitness estimation methods (exposure distributions shown by event status in superimposed histograms).

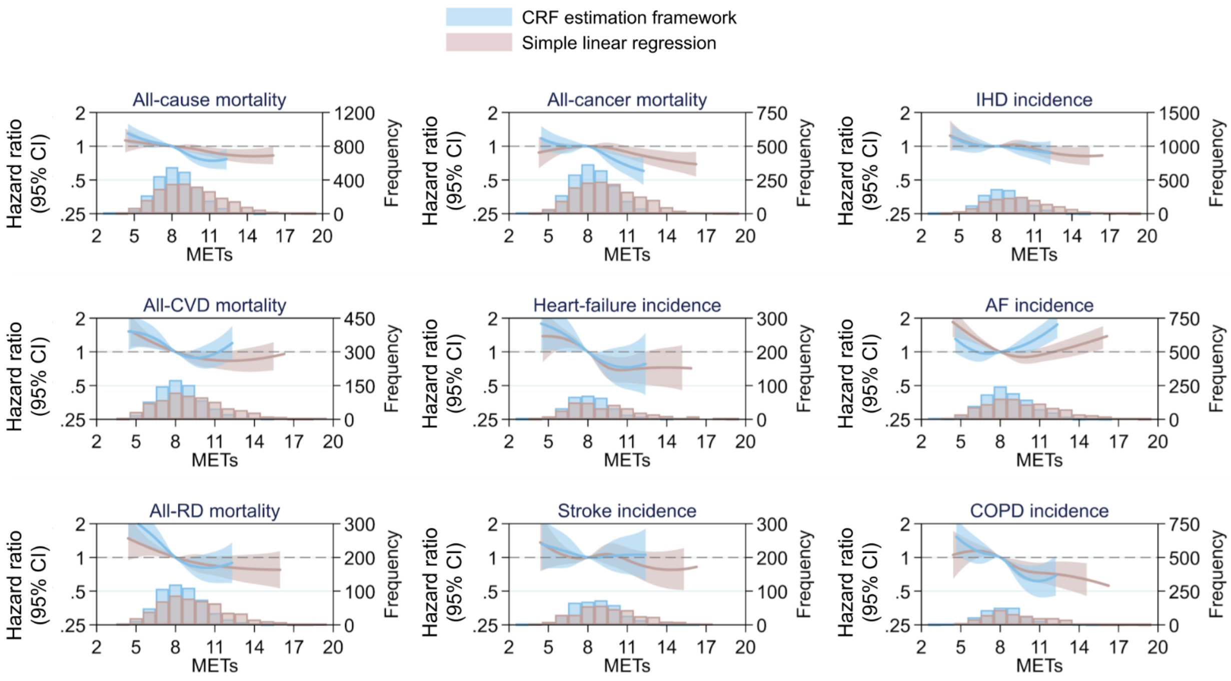

Supplemental Figure 8

Hazard ratios and 95% confidence intervals (CI) for prospective log-linear associations between fatal and non-fatal outcomes in the UK Biobank with cardiorespiratory fitness in metabolic equivalents (METs, per 3.5 ml $O_2 \cdot kg^{-1} \cdot min^{-1}$) computed when restricting the multilevel framework to a single estimation model. For these analyses, the analytical sample was restricted to those who completed a ramp test. CRF estimates from estimation model M4 were not computed since that level is specific to those who completed a flat test Event rate per 100,000 person-years. AF - atrial fibrillation; COPD: chronic obstructive pulmonary disease; CVD: cardiovascular disease; IHD: ischaemic heart disease; RD- respiratory disease. COPD incidence mostly represents severe COPD since only ~25% of cases end up in hospital.

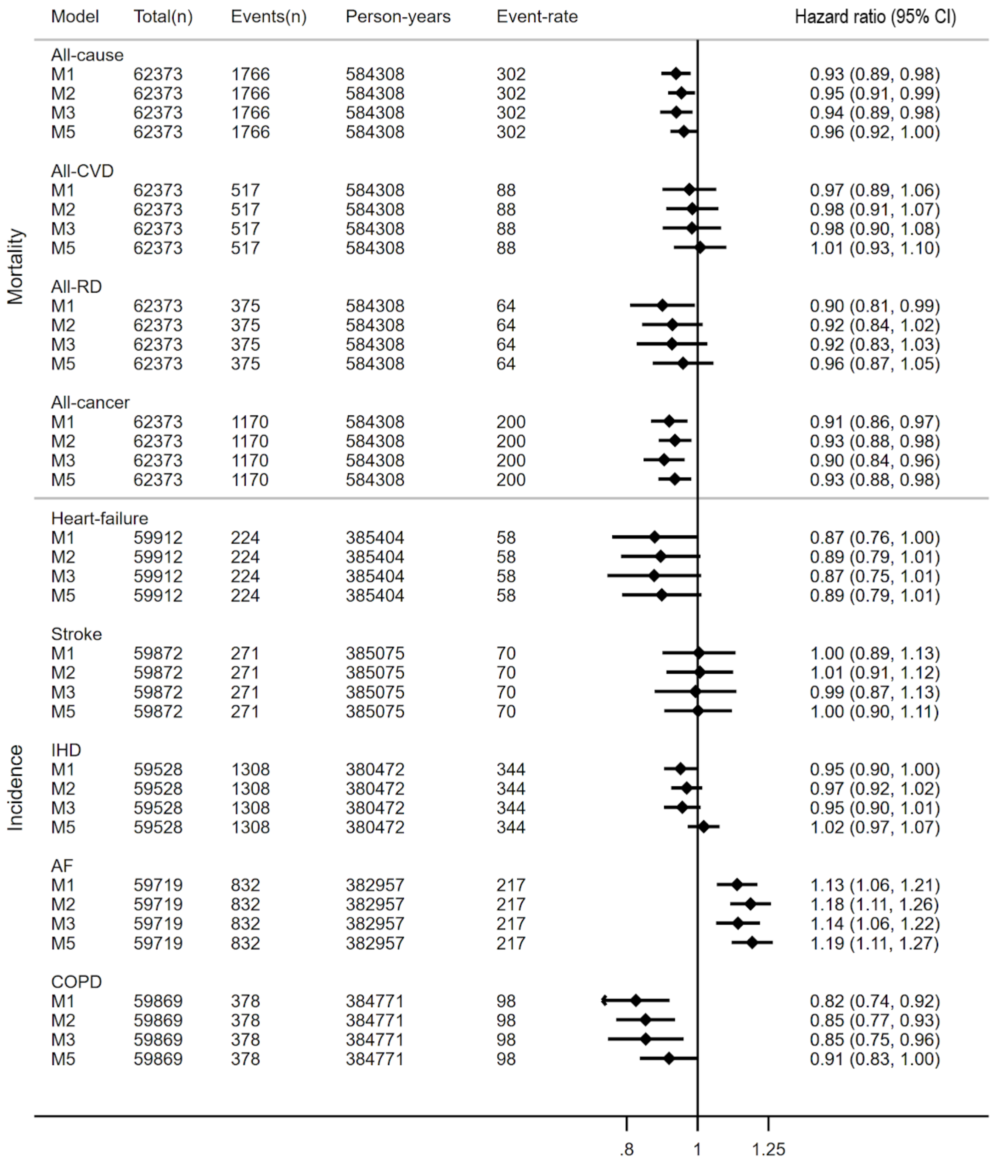

Supplemental Figure 9

Hazard ratios and 95% confidence intervals (CI) for nonlinear (cubic spline) associations between fatal and non-fatal outcomes in the UK Biobank with cardiorespiratory fitness in metabolic equivalents (METs, per 3.5 ml $O_2 \cdot kg^{-1} \cdot min^{-1}$) computed when restricting the multilevel framework to a single estimation model. For these analyses, the analytical sample was restricted to those who completed a ramp test (exposure distributions shown by event status in superimposed histograms). CRF estimates from estimation model M4 were not computed since that level is specific to those who completed a flat test. Hazard ratios were computed relative to a fitness reference point of 8.0 METs. AF: atrial fibrillation; COPD: chronic obstructive pulmonary disease; CVD: cardiovascular disease; IHD: ischaemic heart disease; RD: respiratory disease.

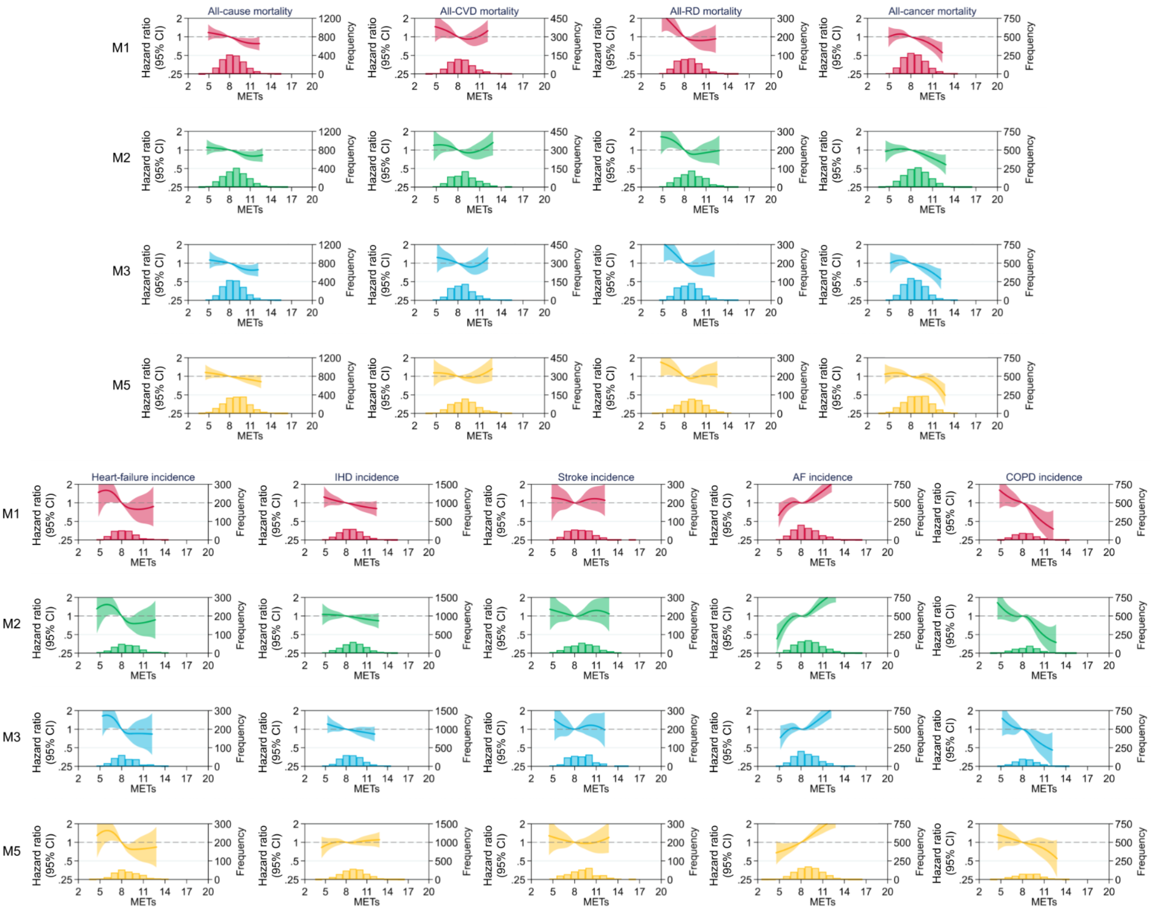

Supplemental Figure 10

Hazard ratios and 95% confidence intervals (CI) for prospective log-linear associations between fatal and non-fatal outcomes in the UK Biobank with cardiorespiratory fitness in metabolic equivalents (METs, per 3.5 ml $O_2 \cdot kg^{-1} \cdot min^{-1}$) computed when restricting the multilevel framework to a single estimation model. For these analyses, the analytical sample was restricted to those who completed a flat test. Event rate per 100,000 person-years. AF - atrial fibrillation; COPD: chronic obstructive pulmonary disease; CVD: cardiovascular disease; IHD: ischaemic heart disease; RD- respiratory disease. COPD incidence mostly represents severe COPD since only ~25% of cases end up in hospital.

	Model	Total(n)	Events(n)	Person-years	Event-rate	Hazard ratio (95%CI)
Mortality	All-cause					
	M4	2333	113	21664	522	1.01 (0.89, 1.15)
	M5	2333	113	21664	522	1.02 (0.90, 1.16)
	All-CVD					
	M4	2333	55	21664	254	0.96 (0.79, 1.17)
	M5	2333	55	21664	254	1.01 (0.84, 1.23)
	All-RD					
	M4	2333	34	21664	157	1.03 (0.82, 1.29)
	M5	2333	34	21664	157	1.04 (0.84, 1.30)
	All-cancer					
	M4	2333	67	21664	309	0.94 (0.79, 1.12)
	M5	2333	67	21664	309	0.94 (0.80, 1.11)
Incidence	Heart-failure					
	M4	1895	15	12121	124	1.03 (0.70, 1.50)
	M5	1895	15	12121	124	1.04 (0.72, 1.49)
	Stroke					
	M4	1889	15	12084	124	1.21 (0.81, 1.81)
	M5	1889	15	12084	124	1.20 (0.82, 1.74)
	IHD					
	M4	1832	110	11503	956	1.06 (0.92, 1.21)
	M5	1832	110	11503	956	1.05 (0.92, 1.19)
	AF					
	M4	1880	55	11956	460	1.06 (0.86, 1.30)
	M5	1880	55	11956	460	1.12 (0.91, 1.36)
	COPD					
	M4	1885	41	11989	342	1.14 (0.91, 1.42)
	M5	1885	41	11989	342	1.10 (0.91, 1.33)

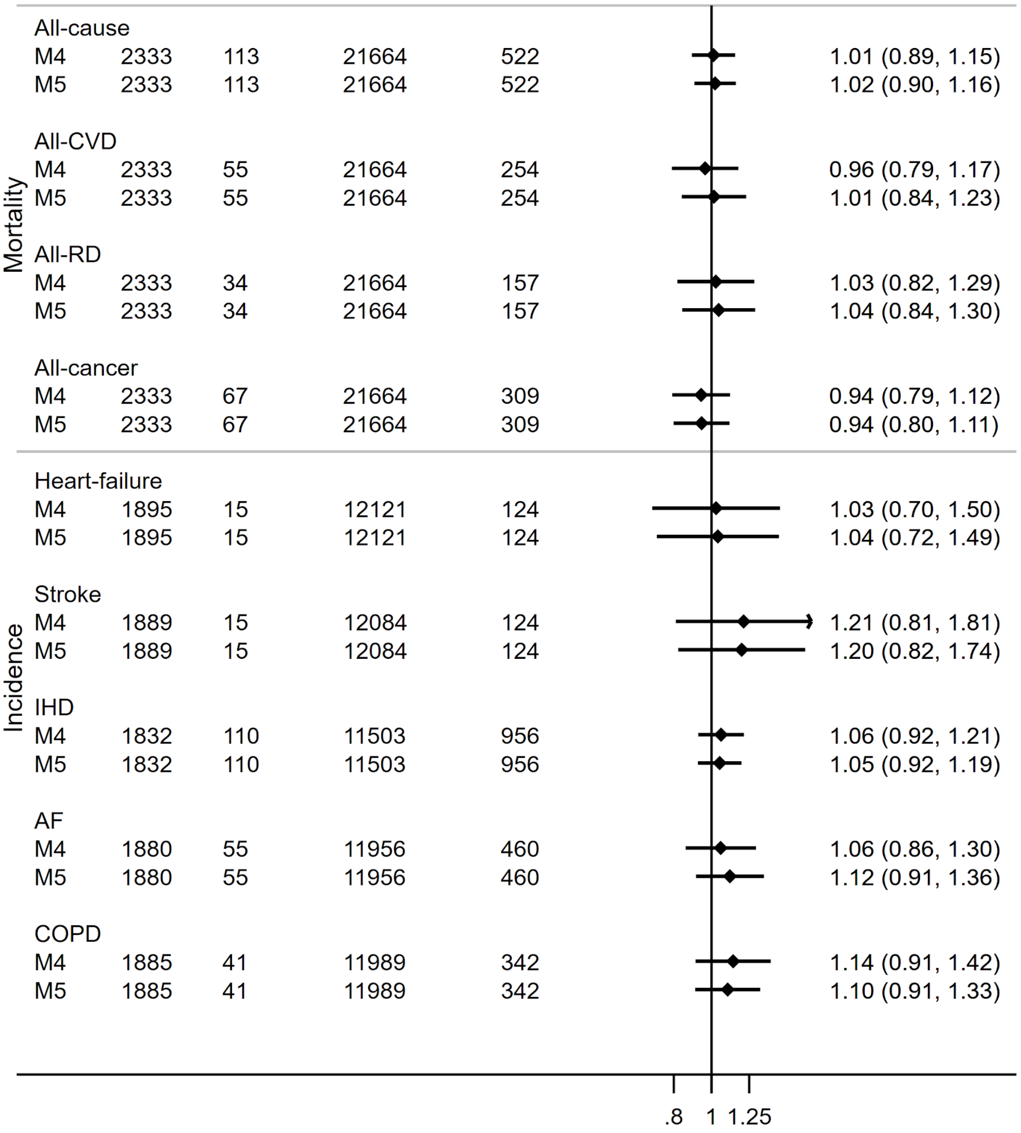

Supplemental Figure 11

Exemplar respiratory exchange data from the ramped maximal exercise test. Work rates corresponding to the lactate threshold (LT) and respiratory compensation point (RCP) were determined by visual inspection of data representing the ventilatory equivalent of oxygen (VE / VO2, lower panel blue dot plot), ventilatory equivalent of carbon dioxide (VE / VCO2, lower panel red dot plot), end-tidal pressure of oxygen (PETO2, upper panel blue dot plot), and end-tidal pressure of carbon dioxide (PETCO2, upper panel red dot plot).

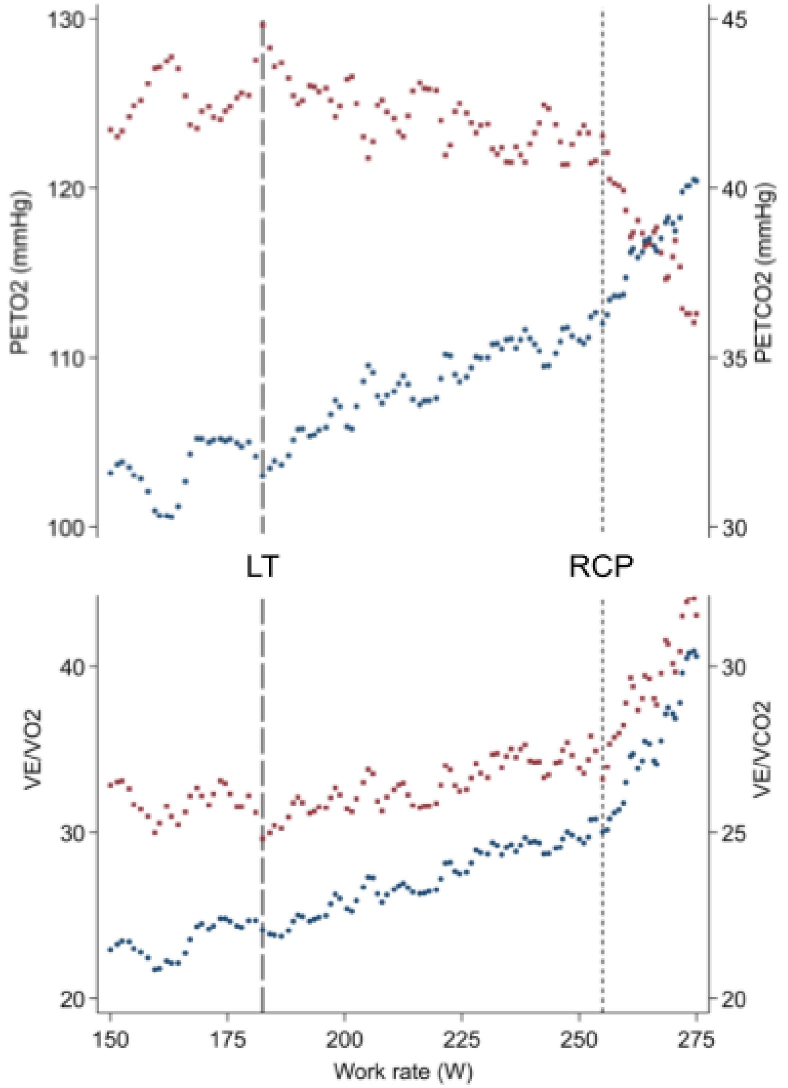

Supplemental Figure 12

Data quality decision diagram for the allocation of multilevel framework estimation models to UKB participants. HR predictions from the ramp phase linear model were solved at the target WR of the UKB CRF test protocol. HR predictions from recovery phase models were solved at T=0s and T=45s. Slope and intercept parameters were defined using the ramp phase linear model. Recovery phase data from the flat protocol (corresponding to equation M4) is not comparable with recovery phase data from ramped protocols (corresponding to equations M1 and M3).

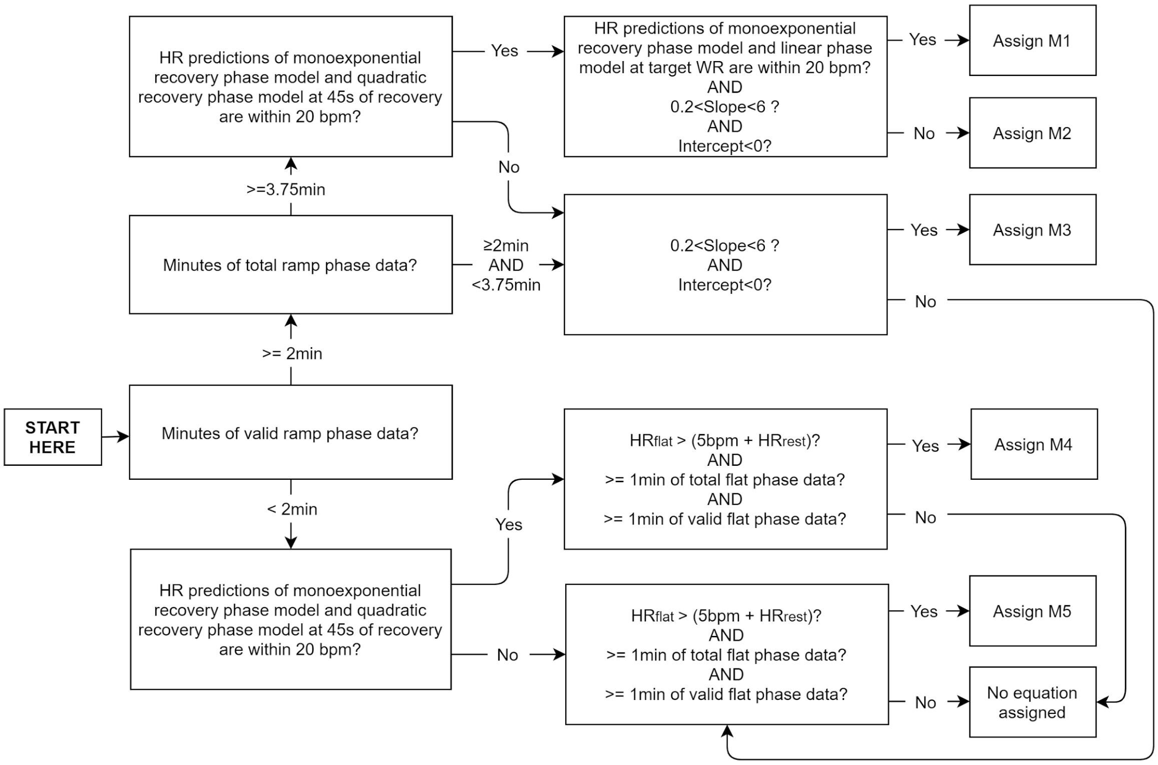

Supplemental Tables

Supplemental Table 1

Descriptions and coefficients for work rate estimation equations at each level of the multilevel framework used to infer cardiorespiratory fitness. The framework has five estimation models (notated as M1-M5), where the top level models (M1) utilises all derived HR features from all stages, while lower level models use progressively fewer features. Thus, the multilevel framework can be adapted to different data availability scenarios. The source of information at each estimation model indicates the UKB CRF test phases used to compute HR features included as predictors at that level. All validation sample participants contributed submaximal exercise test data to the derivation of estimation models. Standard error values for parameter estimates are provided in Supplemental Table 12.

Estimation model	Source of information from UKB fitness test	Work rate estimation equation
M1	Ramp & recovery phases	$-52.8 + 1.44 \cdot HR_{rec45} + 0.562 \cdot b_0 + 14.2 \cdot RR^{0.5} - 1.23 \cdot HR_{rest} - 8.67 \cdot sex + HR_{max} \cdot \left(1.09 - 0.0132 \cdot HR_{rec45} + 0.436 \cdot b_1 - 0.117 \cdot RR^{0.5} + 0.00762 \cdot HR_{rest} + 0.298 \cdot sex\right)$
M2	Recovery phase	$-70.7 + 0.463 \cdot HR_{rec45} + 2.49 \cdot HR_{rec0} - 274 \cdot RR^{0.5} - 3.33 \cdot HR_{rest} - 7.84 \cdot sex + HR_{max} \cdot \left(2.17 - 0.0054 \cdot HR_{rec45} - 0.0249 \cdot HR_{rec0} + 2.78 \cdot RR^{0.5} + 0.0185 \cdot HR_{rest} + 0.363 \cdot sex\right)$
M3	Ramp phase	$-53.1 + 0.621 \cdot b_0 + 87.5 \cdot RR^{0.5} + 0.262 \cdot HR_{rest} - 13.2 \cdot sex + HR_{max} \cdot \left(1.01 + 0.483 \cdot b_1 - 0.791 \cdot RR^{0.5} - 0.00492 \cdot HR_{rest} + 0.327 \cdot sex\right)$
M4	Flat & recovery phases	$-19.5 - 1.31 \cdot HR_{rec45} + 0.434 \cdot HR_{flat} - 44.4 \cdot sex + HR_{max} \cdot \left(2.33 + 0.0151 \cdot HR_{rec45} - 0.0251 \cdot HR_{flat} + 0.674 \cdot sex\right)$
M5	Flat phase	$-10.6 - 0.932 \cdot HR_{rest} - 0.0947 \cdot HR_{flat} - 46.1 \cdot sex + HR_{max} \cdot \left(2.22 + 0.0113 \cdot HR_{rest} - 0.0193 \cdot HR_{flat} + 0.693 \cdot sex\right)$

HR_{max}: Maximal HR (either age-predicted or directly measured), HR_{rest}: Resting HR, HR_{rec45}: Recovery HR at 45s post-exercise, HR_{rec0}: Recovery HR at 0s post-exercise, b_0: Intercept from the ramp phase linear regression model, b_1: Slope from the ramp phase linear regression model, HR_{flat}: Median HR computed for the flat phase, $RR^{0.5}$: Square root of test ramp rate ($W \cdot sec^{-1}$), sex: "0" women,"1" men

Supplemental Table 2

Agreement between work rates measured at the respiratory compensation point (RCP, see Supplemental Figure 11) in the maximal exercise test and work rates computed from flat, low, and high ramp exercise tests using the multilevel framework. M1 results are shown in Figure 2.

Estimation model	Comparator with work rate at RCP	RMSE	*r*	*rho*	Work rate estimation bias (mean ± SD, Watts) Pooled	Females	Males
M1	Estimated WR_{low}	30.5	0.84	0.87	0.6 ± 30.6	2.2 ± 22.0	-0.7 ± 35.8
	Estimated WR_{high}	29.4	0.86	0.87	-1.4 ± 29.4	-1.9 ± 20.7	-1.0 ± 34.7
M2	Estimated WR_{low}	29.3	0.85	0.86	0.3 ± 29.4	3.8 ± 23.0	-2.5 ± 33.4
	Estimated WR_{high}	32.6	0.82	0.83	1.1 ± 32.7	2.2 ± 32.9	0.1 ± 32.7
M3	Estimated WR_{low}	30.8	0.84	0.87	0.4 ± 30.9	1.7 ± 21.3	-0.5 ± 36.6
	Estimated WR_{high}	29.3	0.85	0.87	-0.8 ± 29.4	-0.3 ± 19.4	-1.1 ± 35.2
M4	Estimated WR_{flat}	32.3	0.82	0.83	-3.6 ± 32.2	-1.5 ± 26.5	-5.2 ± 36.1
M5	Estimated WR_{flat}	32.9	0.82	0.82	-2.9 ± 32.8	-1.7 ± 27.3	-3.9 ± 36.8
	Estimated WR_{low}	33.6	0.81	0.82	0.2 ± 33.7	2.5 ± 29.6	-1.6 ± 36.6
	Estimated WR_{high}	31.7	0.83	0.84	-3.6 ± 31.6	-3.7 ± 25.3	-3.6 ± 36.1

RCP: Respiratory compensation point, WR_{low}: Work rate computed with features from “low” ramp test, WR_{high}: Work rate computed with features from “high” ramp test, WR_{flat}: Work rate computed with features from “flat” constant-phase test, RMSE: Root-mean-square error, *r* : Pearson’s correlation coefficient, *rho*: Spearman’s rank correlation coefficient, SD: standard deviation, * $p < 0.05$ for one-sample t-test from zero mean bias

Supplemental Table 3

Agreement between work rates measured at the lactate threshold (LT, see Supplemental Figure 11) in the maximal exercise test and work rates computed from flat, low, and high ramp exercise tests using the multilevel framework.

Estimation model	Comparator with work rate at LT	RMSE	*r*	*rho*	Work rate estimation bias (mean ± SD, Watts)		
					Pooled	Females	Males
M1	Estimated WR_{low}	58.0	0.83	0.86	49.8 ± 29.9*	37.1 ± 19.7*	60.5 ± 32.8*
	Estimated WR_{high}	55.9	0.86	0.88	47.4 ± 29.8*	32.6 ± 20.0*	60.0 ± 31.0*
M2	Estimated WR_{low}	59.4	0.80	0.82	50.4 ± 31.4*	40.7 ± 32.5*	59.2 ± 27.9*
	Estimated WR_{high}	62.2	0.77	0.79	50.7 ± 36.1*	39.2 ± 41.0*	61.3 ± 27.1*
M3	Estimated WR_{low}	57.6	0.84	0.86	49.7 ± 29.1*	36.9 ± 18.1*	60.6 ± 32.2*
	Estimated WR_{high}	55.5	0.86	0.88	48.3 ± 27.5*	34.7 ± 16.2*	59.9 ± 29.9*
M4	Estimated WR_{flat}	56.2	0.83	0.84	47.8 ± 29.5*	36.0 ± 24.2*	58.5 ± 30.0*
M5	Estimated WR_{flat}	56.1	0.83	0.83	47.9 ± 29.2*	36.2 ± 23.9*	58.5 ± 29.7*
	Estimated WR_{low}	58.9	0.82	0.83	51.0 ± 29.5*	39.3 ± 26.1*	61.3 ± 28.6*
	Estimated WR_{high}	55.5	0.84	0.85	47.5 ± 28.8*	34.1 ± 22.8*	59.6 ± 28.4*

LT: lactate threshold, WR_{low}: Work rate computed with features from “low” ramp test, WR_{high}: Work rate computed with features from “high” ramp test, WR_{flat}: Work rate computed with features from “flat” constant-phase test, RMSE: Root-mean-square error, *r* : Pearson’s correlation coefficient, *rho*: Spearman’s rank correlation coefficient, SD: standard deviation, * $p < 0.05$ for one-sample t-test from zero mean bias

Supplemental Table 4

Agreement between work rates measured at VO_2max in the maximal exercise test and work rates computed from flat, low, and high ramp exercise tests using the multilevel framework.

Estimation model	Comparator with work rate at exhaustion	RMSE	*r*	*rho*	Work rate estimation bias (mean ± SD, Watts)		
					Pooled	Females	Males
M1	Estimated WR_{low}	47.3	0.79	0.84	-24.3 ± 40.7*	-17.3 ± 24.0*	-29.7 ± 49.3*
	Estimated WR_{high}	47.8	0.80	0.84	-26.7 ± 39.8*	-21.9 ± 22.5*	-30.4 ± 49.1*
M2	Estimated WR_{low}	48.5	0.76	0.81	-23.3 ± 42.7*	-14.0 ± 34.1*	-30.9 ± 47.4*
	Estimated WR_{high}	50.9	0.73	0.77	-22.8 ± 45.6*	-15.9 ± 42.5*	-28.6 ± 47.5*
M3	Estimated WR_{low}	47.6	0.78	0.83	-24.3 ± 41.1*	-17.6 ± 23.8*	-29.3 ± 50.1*
	Estimated WR_{high}	47.4	0.80	0.84	-25.6 ± 40.0*	-20.1 ± 21.7*	-30.0 ± 49.4*
M4	Estimated WR_{flat}	49.1	0.77	0.80	-25.5 ± 42.1*	-18.8 ± 28.4*	-30.9 ± 50.1*
M5	Estimated WR_{flat}	49.3	0.76	0.79	-25.5 ± 42.4*	-18.8 ± 28.5*	-31.0 ± 50.4*
	Estimated WR_{low}	49.2	0.75	0.78	-22.4 ± 43.9*	-15.2 ± 30.3*	-28.1 ± 51.7*
	Estimated WR_{high}	49.1	0.77	0.81	-26.2 ± 41.7*	-20.8 ± 26.2*	-30.5 ± 50.8*

WR_{low}: Work rate computed with features from "low" ramp test, WR_{high}: Work rate computed with features from "high" ramp test, WR_{flat}: Work rate computed with features from "flat" constant-phase test, RMSE: Root-mean-square error, *r* : Pearson's correlation coefficient, *rho*: Spearman's rank correlation coefficient, SD: standard deviation, * $p < 0.05$ for one-sample t-test from zero mean bias

Supplemental Table 5

Agreement between directly measured at VO_2max and VO_2max values computed from different exercise tests and multilevel framework estimation models, using age-predicted maximal HR. M1 results are shown in Figure 3.

Estimation model	Comparator with measured VO_2max	RMSE	*r*	*rho*	VO_2max estimation bias (mean ± SD, ml $O_2 \cdot kg^{-1} \cdot min^{-1}$)		
					Pooled	Females	Males
M1	Estimated VO_2max_{low}	4.9	0.70	0.74	0.1 ± 4.9	-0.1 ± 4.4	0.2 ± 5.3
	Estimated VO_2max_{high}	4.8	0.72	0.74	-0.2 ± 4.8	-0.6 ± 4.4	0.0 ± 5.1
M2	Estimated VO_2max_{low}	4.5	0.74	0.74	-0.1 ± 4.6	-0.1 ± 4.6	-0.1 ± 4.5
	Estimated VO_2max_{high}	4.7	0.73	0.72	-0.2 ± 4.7	-0.8 ± 4.8	0.2 ± 4.6
M3	Estimated VO_2max_{low}	5.0	0.68	0.74	0.0 ± 5.0	-0.3 ± 4.4	0.3 ± 5.5
	Estimated VO_2max_{high}	4.8	0.70	0.73	-0.1 ± 4.8	-0.5 ± 4.4	0.1 ± 5.2
M4	Estimated VO_2max_{flat}	5.0	0.68	0.68	-0.3 ± 5.0	-0.4 ± 5.1	-0.2 ± 4.9
M5	Estimated VO_2max_{low}	4.9	0.69	0.68	-0.3 ± 4.9	-0.3 ± 5.0	-0.3 ± 4.9
	Estimated VO_2max_{high}	4.8	0.70	0.70	0.2 ± 4.9	0.4 ± 4.9	-0.1 ± 4.9
	Estimated VO_2max_{flat}	4.8	0.71	0.70	-0.3 ± 4.8	-0.2 ± 4.8	-0.3 ± 4.8

VO_2max: Maximal oxygen consumption, VO_2max_{low}: VO_2max computed with features from "low" ramp test, VO_2max_{high}: VO_2max computed with features from "high" ramp test, VO_2max_{flat}: VO_2max computed with features from "flat" constant-phase test, RMSE: Root-mean-square error, *r* : Pearson's correlation coefficient, *rho*: Spearman's rank correlation coefficient, SD: standard deviation, * $p < 0.05$ for one-sample t-test from zero mean bias

Supplemental Table 6

Agreement between directly measured at VO_2max and VO_2max values estimated from different exercise tests and multilevel framework estimation models, using directly measured maximal HR.

Estimation model	Comparator with measured VO_2max	RMSE	*r*	*rho*	VO_2max estimation bias (mean ± SD, ml $O_2 \cdot kg^{-1} \cdot min^{-1}$)		
					Pooled	Females	Males
M1	Estimated VO_2max_{low}	4.8	0.71	0.74	0.3 ± 4.8	0.3 ± 4.2	0.3 ± 5.2
	Estimated VO_2max_{high}	4.4	0.75	0.75	-0.1 ± 4.5	-0.2 ± 4.3	0.0 ± 4.6
M2	Estimated VO_2max_{low}	4.8	0.70	0.69	0.2 ± 4.8	0.3 ± 4.5	0.1 ± 5.1
	Estimated VO_2max_{high}	4.7	0.72	0.70	-0.1 ± 4.7	-0.5 ± 4.5	0.3 ± 4.8
M3	Estimated VO_2max_{low}	4.9	0.69	0.71	0.3 ± 4.9	0.1 ± 4.2	0.4 ± 5.4
	Estimated VO_2max_{high}	4.5	0.74	0.74	0.1 ± 4.6	-0.1 ± 4.3	0.2 ± 4.8
M4	Estimated VO_2max_{flat}	4.9	0.69	0.67	0.0 ± 4.9	0.0 ± 4.8	0.0 ± 5.1
M5	Estimated VO_2max_{low}	4.9	0.69	0.67	0.0 ± 4.9	0.1 ± 4.7	0.0 ± 5.1
	Estimated VO_2max_{high}	5.0	0.68	0.68	0.5 ± 5.0	0.9 ± 4.7	0.1 ± 5.3
	Estimated VO_2max_{flat}	5.0	0.68	0.67	0.0 ± 5.0	0.2 ± 4.7	-0.1 ± 5.3

VO_2max: Maximal oxygen consumption, VO_2max_{low}: VO_2max computed with features from “low” ramp test, VO_2max_{high}: VO_2max computed with features from “high” ramp test, VO_2max_{flat}: VO_2max computed with features from “flat” constant-phase test, RMSE: Root-mean-square error, *r* : Pearson’s correlation coefficient, *rho*: Spearman’s rank correlation coefficient, SD: standard deviation, * $p < 0.05$ for one-sample t-test from zero mean bias

Supplemental Table 7

Internal agreement between VO_2max estimated within each level of the multilevel framework. M1-M3 and M5 were compared when using features computed from ramp tests, and M4 and M5 were compared when using features computed from flat tests. Bias values were computed as the difference between the first and second comparators.

First comparator	Second comparator	RMSE	*r*	*rho*	VO_2max estimation bias (mean ± SD, ml $O_2 \cdot kg^{-1} \cdot min^{-1}$)		
					Pooled	Females	Males
VO_2max_{high} from M1	VO_2max_{low} from M1	2.2	0.94	0.94	-0.4 ± 2.2*	-0.5 ± 2.3	-0.2 ± 1.9
VO_2max_{high} from M2	VO_2max_{low} from M2	3.1	0.96	0.96	0.0 ± 3.1	-0.7 ± 2.0*	0.2 ± 1.1
VO_2max_{high} from M3	VO_2max_{low} from M3	1.9	0.94	0.95	-0.2 ± 1.9	-0.2 ± 1.8	-0.2 ± 1.9
VO_2max_{flat} from M4	VO_2max_{flat} from M5	0.9	0.99	0.99	-0.0 ± 0.9	-0.1 ± 0.8	0.0 ± 0.9
VO_2max_{flat} from M4	VO_2max_{low} from M1	2.7	0.91	0.92	-0.2 ± 2.7	-0.3 ± 2.4	-0.2 ± 2.8
VO_2max_{flat} from M4	VO_2max_{high} from M1	2.5	0.92	0.92	0.2 ± 2.5	0.5 ± 2.6	0.0 ± 2.4
VO_2max_{high} from M5	VO_2max_{low} from M5	1.9	0.98	0.98	-0.6 ± 1.8*	-0.6 ± 1.0*	-0.3 ± 0.9*

VO_2max: Maximal oxygen consumption, VO_2max_{low}: VO_2max computed with features from "low" ramp test, VO_2max_{high}: VO_2max computed with features from "high" ramp test, VO_2max_{flat}: VO_2max computed with features from "flat" constant-phase test, RMSE: Root-mean-square error, *r* : Pearson's correlation coefficient, *rho*: Spearman's rank correlation coefficient, SD: standard deviation, * $p < 0.05$ for one-sample t-test from zero mean bias

Supplemental Table 8

Characteristics of UK Biobank participants in the subsample with cycle ergometer data. Percentages were calculated relative to totals for each sex separately.

Characteristics	Women (42,535)	Men (37,746)
Ethnicity, % (N)		
White	92% (39,048)	93% (34,705)
Mixed, Asian, and Black	8% (3,263)	7% (2,787)
Employment, % (N)		
Unemployed	43% (18,015)	39% (14,482)
Employed	57% (24,300)	61% (23,031)
Townsend deprivation index	-1.3 ± 2.9	-1.3 ± 3.0
Smoking, % (N)		
Never	61% (25,999)	51% (19,290)
Previous	31% (13,287)	38% (14,376)
Current	7% (3,053)	10% (3,872)
Mixed, Asian, and Black	8% (3,263)	7% (2,787)
Alcohol consumption, % (N)		
Never	5% (2,294)	3% (1,059)
Previous	3% (1,274)	3% (1,139)
Current	53% (22,306)	42% (15,677)
Current, three or more per week	39% (16,556)	53% (19,770)
Red or processed meat intake (days/week)	0.8 ± 0.5	1.0 ± 0.6
Weight (kg)	70 ± 13	85 ± 14
Obesity prevalence (BMI > 30kg·m^{-2}), % (N)	20% (8694)	23% (8845)
Disease prevalence at baseline, % (N)		
Hypertension	45% (18,966)	59% (22,249)
Diabetes	3% (1,362)	6% (2,397)
All-cause cancer	15% (6,271)	10% (3,639)
Heart-failure	≤1% (41)	≤1% (159)
Ischaemic heart disease	2% (668)	5% (1958)
Stroke	≤1% (152)	≤1% (258)
Atrial fibrillation	≤1% (229)	2% (660)
Chronic obstructive pulmonary disease	≤1% (132)	≤1% (175)
Medication use, % (N)		
Beta blockers	4% (1,897)	7% (2,647)
Calcium channel blockers	5% (2,146)	9% (3,553)
ACE inhibitors	9% (3,965)	17% (6,490)
Diuretics	6% (2,713)	7% (2,478)
Bronchodilators	7% (2,993)	6% (2,289)
Lipid-lowering agents	12% (5,073)	24% (8,911)
Iron deficiency agents	≤1% (631)	≤1% (323)

Supplemental Table 9

UKB participant characteristics by tertile of cardiorespiratory fitness when computed from the multilevel framework.

Sex	Women						Men					
CRF Tertiles	Lower		Middle		Higher		Lower		Middle		Higher	
	N	VO_2max	N	VO_2max	N	VO_2max	N	VO_2max	N	VO_2max	N	VO_2max
Age group(y)												
Younger than 50	3,029	21.2 ± 3.1	3,030	27.1 ± 1.3	3,029	33.6 ± 3.4	2,541	27.9 ± 2.7	2,542	33.9 ± 1.5	2,541	40.6 ± 3.4
50-54	2,176	20.5 ± 3.0	2,178	26.1 ± 1.3	2,176	32.3 ± 3.4	1,686	26.9 ± 2.7	1,687	32.8 ± 1.4	1,686	39.7 ± 3.4
55-59	2,536	20.0 ± 2.9	2,537	25.2 ± 1.2	2,536	31.1 ± 3.3	2,001	26.2 ± 2.7	2,002	32.1 ± 1.4	2,001	38.6 ± 3.6
60-64	3,487	19.1 ± 3.0	3,489	24.1 ± 1.1	3,487	29.6 ± 3.2	3,091	25.6 ± 2.5	3,093	31.2 ± 1.3	3,091	37.4 ± 3.4
65 and older	2,887	18.2 ± 3.0	2,887	23.2 ± 1.0	2,887	28.3 ± 3.0	3,229	24.8 ± 2.6	3,230	30.0 ± 1.3	3,229	36.1 ± 3.4
Combined across age groups	14,115	19.8 ± 3.2	14,121	25.1 ± 1.8	14,115	30.9 ± 3.8	12,548	26.1 ± 2.9	12,554	31.8 ± 2.0	12,548	38.2 ± 3.8
Age (y)	57 ± 8		57 ± 8		57 ± 8		58 ± 8		58 ± 8		58 ± 8	
Height (cm)	163 ± 6		163 ± 6		163 ± 6		176 ± 7		176 ± 7		175 ± 7	
Weight (kg)	80 ± 15		70 ± 10		63 ± 8		94 ± 14		85 ± 11		77 ± 10	
Body mass index ($kg \cdot m^{-2}$)	30.1 ± 5.3		26.2 ± 3.4		23.5 ± 2.8		30.4 ± 4.1		27.3 ± 2.9		25.1 ± 2.7	
Fat-free mass (kg)	46 ± 5		44 ± 4		43 ± 4		67 ± 8		63 ± 7		60 ± 7	
VO_2max per kg fat-free mass ($ml \cdot kg^{-1} \cdot min^{-1}$)	33.7 ± 5.7		39.6 ± 3.7		45.3 ± 5.1		36.8 ± 3.9		42.6 ± 3.1		48.8 ± 4.5	
Resting blood pressure (mmHg)												
Systolic	135 ± 17		130 ± 17		125 ± 17		139 ± 16		136 ± 15		132 ± 15	
Diastolic	82 ± 9		78 ± 9		74 ± 9		84 ± 9		81 ± 9		78 ± 9	
Resting HR (bpm)	73 ± 10		66 ± 8		62 ± 8		73 ± 11		64 ± 9		59 ± 8	
FVC (L)	3.0 ± 0.6		3.2 ± 0.6		3.3 ± 0.6		4.2 ± 1.0		4.4 ± 0.9		4.5 ± 0.9	
FEV1 (L)	2.3 ± 0.5		2.4 ± 0.5		2.5 ± 0.5		3.2 ± 0.7		3.3 ± 0.7		3.4 ± 0.7	
PEF (L/min)	335 ± 84		342 ± 81		343 ± 82		480 ± 123		492 ± 122		496 ± 118	
Smoking status, % (N)												
Never	64% (8,947)		61% (8,603)		59% (8,330)		48% (5,977)		51% (6,318)		55% (6,932)	
Previously	29% (4,119)		32% (4,441)		33% (4,675)		41% (5,163)		39% (4,853)		35% (4,333)	
Currently	7% (967)		7% (1,010)		8% (1,063)		11% (1,319)		10% (1,317)		10% (1,230)	
Health self-rating, % (N)												
Excellent	9% (1,228)		16% (2,272)		25% (3,487)		8% (989)		14% (1,731)		23% (2,929)	
Good	60% (8,493)		66% (9,241)		63% (8,866)		53% (6,671)		62% (7,814)		61% (7,695)	
Fair	27% (3,727)		16% (2,288)		11% (1,538)		33% (4,150)		21% (2,626)		14% (1,700)	
Poor	4% (573)		2% (257)		1% (180)		6% (650)		3% (318)		2% (192)	

Values are means ± standard deviations, unless otherwise indicated. CRF: Cardiorespiratory fitness, VO_2max: Maximal oxygen consumption ($ml \cdot kg^{-1} \cdot min^{-1}$) , HR: Heart rate, FVC: Forced vital capacity, FEV1: Forced expiratory volume (1s), PEF: Peak expiratory flow

Supplemental Table 10

Sampling strata for validation study participants. Participants were selected using a stratified random sampling procedure for which the strata were sex, age (40-49y, 50-59y, 60-69y), and BMI. The range of each BMI strata covered at least the 25th and 75th percentile in the equivalent age/sex strata in the UKB sample, aiming to ensure that the validation study sample was broadly representative of fitness levels across strata in the UKB cohort.

Age range (y)	40-49		50-59		60-69	
Sex	F	M	F	M	F	M
BMI group 1	20.5-23.9	22.0-25.4	21.0-23.9	22.5-25.4	21.5-24.4	22.9-25.8
BMI group 2	24.0-27.4	25.5-28.4	24.0-27.4	25.5-28.9	24.5-28.4	25.9-28.9
BMI group 3	27.5-35.0	28.5-33.5	27.5-35.0	29.0-34.0	28.5-34.5	29.0-33.5

F: Female, M: Male, BMI: Body mass index ($kg \cdot m^{-2}$)

Supplemental Table 11

Overview of tests completed by validation study participants; tests were parameterised according to the participant's individualised UKB protocol. For example, a male participant with UKB test "M100" completed a flat test at 40W, two ramped tests with target WR values of 100W and 130W, a steady-state test, and a ramped VO2max test. Flat tests consisted of one steady-state work rate for 6 minutes. Ramped tests consisted of an initial steady-state WR for 2 minutes and incremented at a rate equal to RR for 4 minutes until the target WR was reached. Steady-state tests consisted of four consecutive steady-state work rates (WR1-4) at 4 minutes each. Maximal ramped tests consisted of an initial WR and incremented at a rate equal to RR until exhaustion.

UKB allocation	Flat test	Low ramped test			High ramped test			Steady-state test				Ramped VO_2max test	
	WR	Initial WR	Target WR	RR	Initial WR	Target WR	RR	WR 1	WR 2	WR 3	WR 4	Initial WR	RR
F30	30	30	50	5	30	80	12.5	45	55	65	75	65	15
F40	30	30	50	5	30	80	12.5	45	55	65	75	65	15
F50	30	30	50	5	30	80	12.5	45	55	65	75	65	15
F60	30	30	60	7.5	30	90	15	45	55	65	75	65	15
F70	30	30	70	10	30	100	17.5	45	55	65	75	65	15
F80	30	30	80	12.5	30	100	17.5	45	55	65	75	65	15
F90	30	30	60	7.5	30	90	15	45	60	75	90	75	20
F100	30	30	70	10	30	100	17.5	45	60	75	90	75	20
F110	30	30	70	10	30	110	17.5	45	60	75	90	75	20
F120	30	30	70	10	30	110	20	45	60	75	90	75	20
F130	30	30	70	10	30	110	20	45	60	75	90	75	20
M40	40	40	70	7.5	40	110	17.5	60	75	90	105	90	20
M50	40	40	70	7.5	40	110	17.5	60	75	90	105	90	20
M60	40	40	70	7.5	40	110	17.5	60	75	90	105	90	20
M70	40	40	70	7.5	40	110	17.5	60	75	90	105	90	20
M80	40	40	80	10	40	120	20	60	75	90	105	90	20
M90	40	40	90	12.5	40	130	22.5	60	75	90	105	90	20
M100	40	40	100	15	40	130	22.5	60	75	90	105	90	20
M110	40	40	80	10	40	110	17.5	60	80	100	120	100	30
M120	40	40	90	12.5	40	120	20	60	80	100	120	100	30
M130	40	40	100	15	40	130	22.5	60	80	100	120	100	30
M140	40	40	100	15	40	140	25	60	80	100	120	100	30

UKB: UK Biobank, F: Female, M: Male, WR: Work rate (W), RR: Ramp rate ($W \cdot min^{-1}$)

Supplemental Table 12

Model parameters, parameter estimates, and standard error values for each estimation model (notated as M1-M5) of the multilevel framework.

	Estimation Model				
	M1	M2	M3	M4	M5
Model Parameter					
HR_{ss}	1.09** (0.10)	2.17** (0.10)	1.01** (0.10)	2.33** (0.15)	2.22** (0.15)
Intercept	-52.8** (14.4)	-70.7** (16.5)	-53.1** (14.8)	-19.5 (22.0)	-10.6 (21.4)
b_0	0.562** (0.023)		0.621** (0.024)		
$b_1 \cdot HR_{ss}$	0.436** (0.015)		0.483** (0.015)		
$RR^{0.5}$	14.2 (18.0)	-274** (24)	87.5** (17.3)		
$RR^{0.5} \cdot HR_{ss}$	-0.117 (0.144)	2.78** (0.18)	-0.791** (0.139)		
HR_{rec0}		2.49** (0.18)			
$HR_{rec0} \cdot HR_{ss}$		-0.0249** (0.0013)			
HR_{rec45}	1.44** (0.15)	0.463* (0.18)		-1.31 (0.58)	
$HR_{rec45} \cdot HR_{ss}$	-0.0132** (0.0011)	-0.0054** (0.0014)		0.0151** (0.0039)	
HR_{flat}				0.434 (0.596)	-0.0947 (0.411)
$HR_{flat} \cdot HR_{ss}$				-0.0251** (0.0040)	-0.0193** (0.0028)
HR_{rest}	-1.23** (0.24)	-3.33** (0.26)	0.262 (0.19)		-0.932 (0.441)
$HR_{rest} \cdot HR_{ss}$	0.00762** (0.00146)	0.0185** (0.0016)	-0.00492** (0.00108)		0.0113** (0.0030)
sex	-8.67* (3.71)	-7.84 (4.61)	-13.2** (3.7)	-44.4** (5.2)	-46.1** (5.0)
$sex \cdot HR_{ss}$	0.298** (0.024)	0.363** (0.026)	0.327** (0.025)	0.674** (0.035)	0.693** (0.034)

Values are model coefficients (standard error). HR_{ss}: Steady-state HR (Note that maximal HR may be substituted for HR_{ss} to estimate VO_2max), HR_{rest}: Resting HR, HR_{rec45}: Recovery HR at 45s post-exercise, HR_{rec0}: Recovery HR at 0s post-exercise, b_0: Intercept from the ramp phase linear regression model, b_1: Slope from the ramp phase linear regression model, HR_{flat}: Median HR computed for the flat phase, $RR^{0.5}$: Square root of test ramp rate ($W \cdot sec^{-1}$), sex: "0" women,"1" men, *Intercept*: Model intercept, *: $p < 0.05$, **: $p < 0.01$
